# Percutaneous Insertion of Hepatic Arterial Infusion Ports by Interventional Radiology for Colorectal Liver Metastases

**DOI:** 10.3390/cancers18213495

**Published:** 2026-10-29

**Authors:** Hugo C. Temperley, Omar Abu Saadeh, Padraig Cronin, Cormac B. Mulhall, Niall J. O’Sullivan, Benjamin M. Mac Curtain, Alice Talbot, Emily Harrold, David Gallagher, Michael E. Kelly, Hooman Yarmohammadi, Vlasios S. Sotirchos

**Affiliations:** 1 Department of Radiology, St James’s Hospital, D08 NHY1 Dublin, Ireland; 2 Trinity St James Cancer Institute, St James’s Hospital, D08 NHY1 Dublin, Ireland; kellym11@tcd.ie; 3 Royal College of Surgeons in Ireland, D02 YN77 Dublin, Ireland; 4 Department of Surgery, Cork University Hospital, T12 DC4A Cork, Ireland; 5 Department of General Surgery, Royal Perth Hospital, Perth, WA 6000, Australia; 6 Northwell Health, New Hyde Park, NY 11040, USA; 7 Department of Urology, Lenox Hill Hospital, New York, NY 10075, USA; 8 Department of Medical Oncology, St James’s Hospital, D08 NHY1 Dublin, Ireland; alicetalbot@alumnircsi.com (A.T.);; 9 Department of Genetics, St James’s Hospital, D08 NHY1 Dublin, Ireland; 10 Department of Surgery, University of Dublin, Trinity College Dublin, D02 PN40 Dublin, Ireland; 11 Department of Radiology, Memorial Sloan Kettering Cancer Center, 1275 York Avenue, New York, NY 10065, USAsotirchv@mskcc.org (V.S.S.)

**Keywords:** hepatic arterial infusion, colorectal liver metastases, interventional radiology, port–catheter system, percutaneous implantation, technical success, device failure, meta-analysis

## Abstract

**Background:** Hepatic arterial infusion (HAI) chemotherapy for colorectal liver metastases (CRLM) is delivered through a port–catheter system implanted percutaneously by interventional radiology or surgically. Previous studies have pooled device complications across access methods as event counts, but have not synthesised the technical performance and durability of fully percutaneous insertion at the patient level. **Methods:** Medline, EMBASE, Cochrane Library, Scopus and Web of Science were searched for studies published from 2000 onward reporting adults with CRLM undergoing HAI port insertion. Primary analyses were restricted to fully percutaneous implanted ports, using outcome-specific, patient-level numerators and denominators; a study using surgical arterial cutdown was added in sensitivity analyses. Risk of bias in the device outcomes was assessed at item level for patient selection, completeness of data, outcome ascertainment and selective reporting, with Cochrane RoB 2 used additionally for the single randomised trial. Random-effects proportion meta-analyses used the Freeman–Tukey double arcsine transformation and the DerSimonian–Laird estimator, with a binomial generalised linear mixed model (GLMM) as an alternative. Outcomes with inconsistent definitions or denominators were synthesised narratively. **Results:** Fifteen studies contributed 811 patients, 654 of whom underwent percutaneous insertion. One study was randomised, five prospective and nine retrospective; three reported a surgical comparator arm. Risk of bias in the device outcomes was low in one study, moderate in six and high in eight. Eleven studies (423 patients) of fully percutaneous implanted ports contributed to the primary analyses. Technical success was reported explicitly by eight and achieved in 276 of 278 patients (99.3%, exact 95% CI 97.4–99.9%); with only two failures, pooled models added little. Device-attributable definitive failure, meaning permanent discontinuation of HAI because of the device, occurred in 39 of 374 patients (crude 10.4%) over observation periods that varied widely; the random-effects estimate was 6.9% (95% CI 2.1–13.7%; I^2^ = 74.5%; GLMM 6.9%, 3.0–15.2%), and 11.6% (95% CI 6.3–18.0%) in the six studies at low or moderate risk of bias. Catheter dislodgement affected a pooled 5.8% (95% CI 1.6–11.6%), and 14.8% where the port was checked angiographically before every course or every four weeks; hepatic arterial occlusion affected 3.4% (95% CI 0.9–7.0%; four studies). Revision was required in 56% and 59% of percutaneous patients in the two series reporting it. **Conclusions:** Percutaneous insertion of HAI ports rarely failed technically in these series, but reintervention was common and permanent loss of HAI to a device problem within the follow-up reported was estimated at 6.9% in the primary analysis and 11.6% in studies at lower risk of bias, with more problems detected where the device was monitored more closely. Catheter-tip technique, follow-up duration and intensity of device surveillance are possible, unproven contributors to heterogeneity. These predominantly observational data support the feasibility of percutaneous access, not patient suitability for HAI chemotherapy.

## Simple Summary

Chemotherapy can be delivered directly into the artery that supplies the liver through a small port placed under the skin, an approach used for bowel cancer that has spread to the liver. The port and its catheter can be inserted by a surgeon during an operation, or by an interventional radiologist through a needle puncture in an artery without open surgery. We combined the published studies of the needle-based approach to find out how often insertion succeeds and how often the device later stops working. Insertion almost always succeeded. However, in some studies more than half of patients needed at least one further procedure to fix the device, and about one in fourteen lost the treatment permanently because of a device problem in the main analysis, or about one in nine when only the more reliable studies were included, with more problems found where the device was checked more often. These findings can help patients and doctors weigh up the needle-based approach and show what better studies need to measure.

## 1. Introduction

Colorectal cancer is among the most commonly diagnosed cancers and a leading cause of cancer-related death worldwide, with an estimated 2.0 million new cases in 2024 [1]. The liver is the most common site of distant spread: up to 70% of patients develop hepatic metastases, and it is the sole metastatic site in roughly one-third of these [2,3]. Only 20–30% of patients with colorectal liver metastases (CRLM) are candidates for curative-intent resection at presentation, and more than half of those resected ultimately recur [2].

For patients who are not resection candidates, and increasingly as an adjunct after resection, regional liver-directed therapy has an established role alongside systemic chemotherapy. International oncology guidelines diverge on the optimal choice and timing of local therapies, including selective internal radiation therapy (SIRT), stereotactic radiation and hepatic arterial infusion (HAI), reflecting variability in patient populations and limited availability of the relevant specialist services [3,4,5]. HAI exploits the liver’s dual blood supply: whereas normal hepatocytes derive most of their perfusion from the portal vein, liver metastases derive up to 80–90% of their blood supply from the hepatic artery [6]. Direct arterial delivery therefore achieves markedly higher tumour drug concentrations and correspondingly lower systemic exposure than intravenous administration of the same agent. The agent used most often is floxuridine, whose high (>95%) hepatic first-pass extraction provides a strong pharmacological rationale for arterial delivery, with fluorouracil and, more recently in Europe, oxaliplatin-based regimens also in use [6,7,8,9].

The clinical evidence base is substantial. Randomised trials from the 1980s onward showed superior tumour response with hepatic arterial floxuridine than with systemic chemotherapy in unresectable CRLM [10,11,12,13], a response-rate advantage confirmed on pooled analysis, though without a consistent survival benefit against modern comparators [14]; after resection, randomised and propensity-matched data show improved hepatic disease-free and overall survival when HAI is added to systemic chemotherapy [15,16,17]. Consensus guidance accordingly endorses HAI with systemic therapy in selected patients with liver-confined CRLM [18,19], and interest has been renewed over the past decade, including in first-line settings [20,21,22,23,24,25,26].

Delivery of HAI requires a durable catheter–port system positioned so that the whole hepatic arterial territory is perfused while extrahepatic perfusion, and the gastroduodenal ulceration that follows, is avoided, typically by ligating or embolising the gastroduodenal and right gastric arteries around the catheter tip [6]. This was historically achieved only by open surgical placement at laparotomy, which remains standard at many high-volume centres [15,19,20]. Surgical implantation is itself evolving; robotic pump placement is now offered at high-volume centres, and a propensity-matched two-institution series of 819 implantations found it feasible and effective, with shorter length of stay than open placement and no difference in time to starting HAI or in early device failure [27]. The practical comparison over the coming years is therefore likely to be between image-guided and minimally invasive surgical placement rather than between percutaneous and open surgery. Since the late 1990s, percutaneous placement by interventional radiologists, via femoral, subclavian, transaxillary or transbrachial access with fluoroscopically guided embolisation of aberrant arteries, has emerged as the alternative [28,29]; centres have adopted one approach or the other according to local expertise and resources, a divergence compounded by supply disruption affecting both the dedicated surgical pump and the dedicated percutaneous catheter [30,31].

Few single-centre studies have compared the two approaches directly, with conflicting findings: some report more device-related complications after percutaneous placement [32,33], others comparable technical success and complication profiles [34]. Bacchetti et al. pooled device complications in unresectable CRLM by device and access method, reporting 261 complications among 722 patients with radiologically placed catheters [35], but as event counts rather than affected patients and without technical success or device-attributable failure as outcomes; other reviews have addressed the oncological efficacy of HAI [14,21,22] or described technique narratively [28,36,37] rather than pooling performance. This review addresses a narrower question, pooling at patient level technical success together with the device outcomes that determine whether a successfully placed system keeps delivering treatment. Primary analyses were restricted to fully percutaneous implanted ports.

## 2. Methods

### 2.1. Study Design and Reporting Guidelines

This review is reported in line with the PRISMA 2020 guidance for systematic reviews [38], adapted as described in Section 2.5 given the heterogeneous evidence base identified. The protocol was registered prospectively with PROSPERO (CRD420261483144). The population was adults (≥18 years) with colorectal liver metastases undergoing insertion of an HAI device, resectable or unresectable, first-time or revision. The intervention was percutaneous or image-guided insertion; primary analyses were restricted to fully percutaneous arterial access with an implanted port, studies using surgical exposure of the access artery or an external pump contributing to sensitivity analyses or narrative synthesis only; the comparator, where one existed within a study, was surgical insertion, reported descriptively only. Primary outcomes were technical success, device-related complications and need for revision; secondary outcomes were procedure-related morbidity, length of stay, time to chemotherapy initiation, device patency, procedure time, 30-day mortality, cost, conversion rate and, as exploratory outcomes, oncological results. Analysis was restricted to percutaneous insertion throughout, the percutaneous arm of a comparative study contributing on the same basis as a single-arm cohort, so the synthesis is a single-technique proportion meta-analysis rather than a head-to-head comparison.

### 2.2. Search Strategy

**Figure 1:**
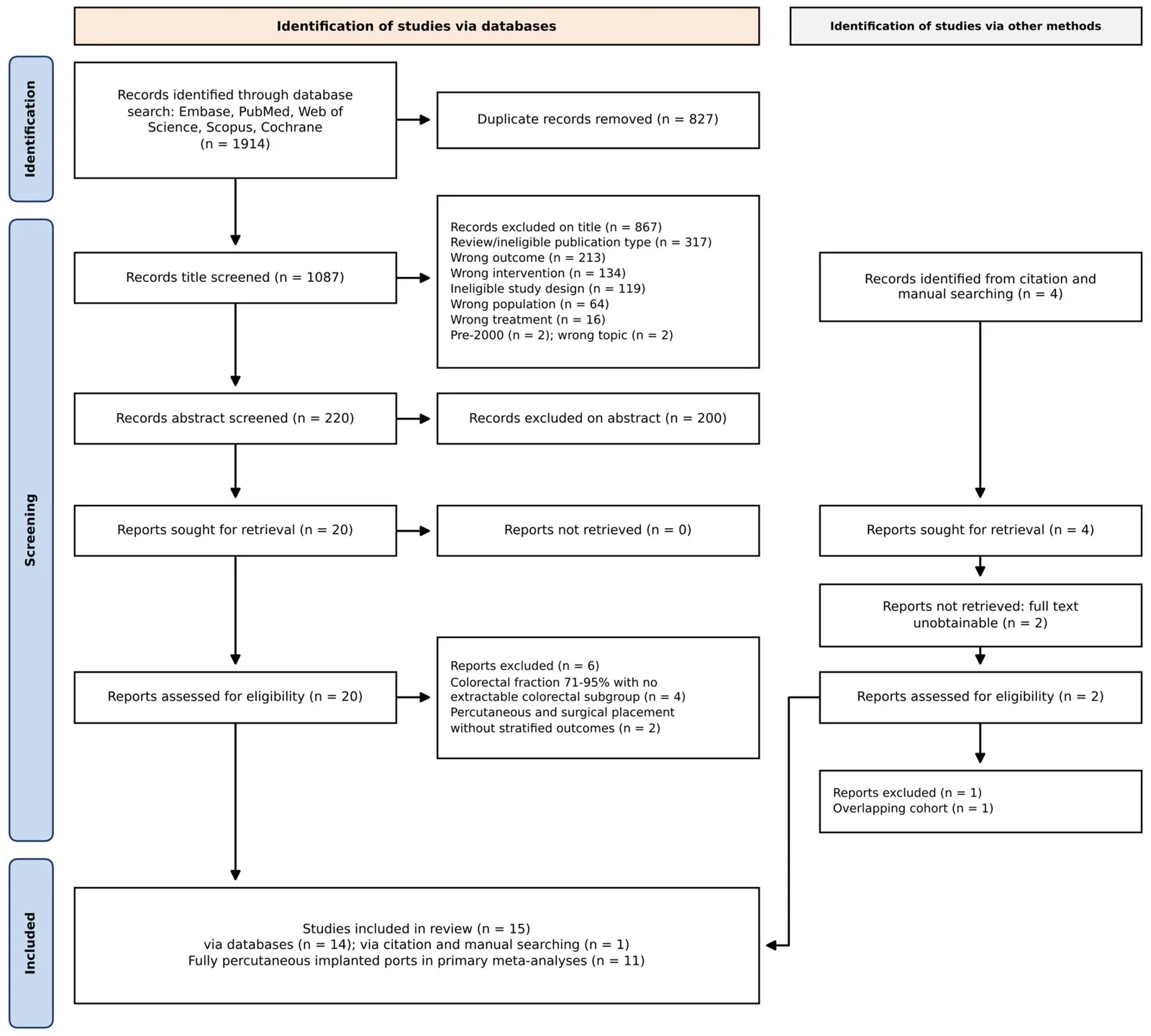
PRISMA 2020 flow chart.

Medline, EMBASE, Cochrane Library, Scopus and Web of Science were searched for studies published from 2000 onward. The full search strategy for each database, with the date of the last search, is provided in Supplementary Material S1. Results were exported to Rayyan [39] for storage and duplicate merging. The databases were last searched on 30 June 2026. In addition, the reference lists of the included full texts and of the percutaneous technique literature were hand-searched and manual searching was undertaken; this identified one further eligible cohort, Deschamps 2011 [34], which had not been retrieved by the database search because it describes the treatment as intra-arterial hepatic chemotherapy rather than hepatic arterial infusion. All records from both sources were assessed against the final eligibility criteria (Section 2.3). Records identified by citation and manual searching are shown in the separate “other methods” arm of the PRISMA flow diagram (Figure 1), which has been corrected in this revision so that each study is counted under the route by which it was identified.

### 2.3. Eligibility Criteria

Eligible designs were randomised trials, prospective or retrospective comparative cohorts, registry studies and single-arm series of *n* ≥ 10, published from 2000 onward in English. Studies had to report an exclusively colorectal-primary population, or a separately extractable colorectal subgroup, and a long-term indwelling arterial device, whether an implanted port or a catheter connected to an external pump, placed percutaneously or under image guidance; devices intended for temporary access were excluded. The 2000 cut-off captures the era in which percutaneous port–catheter placement became an established alternative to surgical implantation [28]. It is a publication-date rather than an enrolment-date limit: one included study reports patients enrolled from 1970 onward [40], so it does not by itself exclude historical technique, as addressed in the limitations.

### 2.4. Study Selection, Data Extraction and Risk of Bias Assessment

Two reviewers (OAS and PC) screened publications independently, with discordance resolved by a third author (HT). Extraction was performed by OAS and independently checked against the source articles by PC, with disagreement again resolved by HT. Study characteristics, insertion technique, access route, catheter-tip fixation method, sample size and all outcomes above were taken directly from the published full text (Table 1). No data were extrapolated beyond what was explicitly reported; numerators and denominators were extracted separately for each outcome at patient level (Supplementary Table S2a). Event counts, such as complications per treatment course, were used only where the number of patients affected could be determined from the source, and technical success was not inferred from the absence of reported failures.

Because the device outcomes in this review are single-arm proportions extracted from the percutaneous arm of each study, risk of bias was assessed for those outcomes specifically, rather than for the comparison each study was designed to make. Four domains, adapted from the JBI critical appraisal checklist for case series [41] and the corresponding domains of ROBINS-I [42], were judged at item level for every study: patient selection (consecutive or complete inclusion, explicit criteria, prospective or retrospective design and whether the cohort was a selected subset of a larger series or was defined by receipt of HAI rather than by implantation); completeness of data (whether every implanted patient was accounted for in the device outcomes); outcome ascertainment (whether the device was checked through the port by angiography or CT angiography before every course or at least four-weekly, at longer scheduled intervals or only at implantation or when a problem was suspected, and whether device outcomes were defined and graded); and selective reporting (whether device outcomes were a stated objective or were reported incidentally). Each domain was judged at low, moderate or high risk, and the overall judgement was the highest risk in any domain. The single randomised trial was additionally assessed with the Cochrane risk-of-bias tool for randomised trials (RoB 2) [43] for its hepatic arterial infusion arm. Assessments were performed independently by OAS and PC, with HT arbitrating. The Newcastle–Ottawa Scale [44] is reported in the Supplementary Material for completeness but was not used to interpret the evidence, since its comparability domain and the credit it gives for a surgical comparator do not bear on the reliability of device outcomes extracted from a single arm.

### 2.5. Statistical Analysis

Complication definitions and denominators (per-patient, per-catheter, per-treatment-course) varied substantially, so a pooled estimate of any device-related complication was judged uninterpretable and is not presented. Pooling was restricted to four outcomes defined consistently wherever reported: technical or primary success; device-attributable definitive failure, meaning permanent discontinuation of HAI because of a device or catheter problem; catheter dislodgement or tip migration; and hepatic arterial occlusion or thrombosis. Only studies reporting the relevant patient-level numerator and denominator explicitly contributed to each estimate. Pooling the three device outcomes is a departure from the protocol, which anticipated pooling technical success alone; all three are named there as review outcomes and met its condition of a consistent definition and denominator, but the decision was taken after the studies were identified. Restriction of the primary analyses to fully percutaneous implanted ports, the patient-level denominator rule and the GLMM were added at peer review.

Single-arm proportion meta-analyses used a random-effects model with Freeman–Tukey double arcsine transformation and the DerSimonian–Laird estimator for tau^2^, back-transformed using the harmonic mean sample size, with heterogeneity quantified by Cochran’s Q and I^2^ [45,46]. Because most technical success proportions lie at the 100% boundary, where back-transformation of the Freeman–Tukey estimate can mislead [47], every analysis was repeated with a binomial–normal random-intercept logistic GLMM fitted by maximum likelihood [48]. The Freeman–Tukey model was retained as primary because it was prespecified in the protocol, the GLMM having been added at peer review. For the device outcomes, whose proportions lie away from the boundary, the two models agreed closely. For technical success they did not: with only two failures among 278 patients, the GLMM interval was far wider than the Freeman–Tukey interval, which reflects the instability of back-transformation near 100% [47] rather than genuine precision, so the crude proportion with an exact Clopper–Pearson 95% CI is reported as the principal summary of technical success. Crude pooled proportions with exact 95% CIs are reported for every analysis (Supplementary Table S2b), because under substantial heterogeneity the random-effects model weights small studies relatively more and its estimate can differ materially from the crude proportion. All GLMMs converged without warnings, except in the two technical-success subgroups in which every study reported 100%, where the model cannot be fitted and is reported as not estimable. Sensitivity analyses, and subgroup analyses of technical success by design and treatment era with between-group heterogeneity tested by Cochran’s Q, are set out in Table 2. At peer review, definitive failure was also re-estimated in the studies at low or moderate overall risk of bias, and dislodgement was compared between studies that checked the port angiographically before every course or at least four-weekly and studies with less frequent or unspecified surveillance; both analyses are post hoc. The one study in which the access artery was surgically exposed was added in a sensitivity analysis for every device outcome. Surgical comparator arms were excluded from all pooled analyses and no between-technique effect estimate was calculated. Analyses were performed in R version 4.3.3 (R Foundation for Statistical Computing, Vienna, Austria) using the meta package version 8.0-1 [49,50], with the GLMM fitted through metafor and lme4; the extracted data and analysis code are provided in Supplementary Table S2a and Supplementary Material S2 and as separate files (HAI_extraction_S3.csv and HAI_meta_analysis_R2.R), which reproduce every estimate reported.

## 3. Results

### 3.1. Included Studies

Fifteen studies met the eligibility criteria and are summarised in Table 1: one randomised controlled trial, of which only the hepatic arterial infusion arm was eligible [51]; five prospective studies [52,53,54,55,56]; and nine retrospective cohorts [34,40,57,58,59,60,61,62,63]. Three reported a percutaneous and a surgical arm separately [34,40,53] and are the only directly comparative studies available in this population; only their percutaneous arms contributed to quantitative synthesis, and no randomised trial of insertion technique was identified. Nine studies were conducted in Japan [51,52,54,55,56,57,58,59,62], two in France [34,63] and one each in the United States [40], Germany [53], Korea [60] and China [61]. Enrolment spanned 1970 to 2022 and all fifteen enrolled exclusively colorectal-primary populations. In total 811 patients contributed, 654 in percutaneous arms or cohorts (range 10–135) and 157 in the surgical comparator arms [34,40,53]. Eleven studies (423 patients) used fully percutaneous arterial access with an implanted port and contributed to at least one primary analysis [34,52,53,54,55,56,57,59,60,61,63]. Of the prespecified secondary outcomes, device patency, procedure-related morbidity and oncological outcomes were reported by most studies and conversion to resectability by two [58,61]; length of stay, time to chemotherapy initiation, 30-day mortality and cost were reported by none. Figure 1 shows the PRISMA 2020 flow diagram. Fourteen included studies were identified through the database search and one, Deschamps 2011 [34], through citation searching.

**Table 1:** Characteristics of the fifteen included studies.

Study	Country	Design	Access Route and Catheter Technique	*n*	Setting and Regimen
Oberfield 2004 [40]	USA	Retrospective comparative cohort, 1970–1995	Transbrachial; external portable pump (*n* = 42) vs. surgical (*n* = 58)	42 (+58 surgical)	Unresectable; FUDR/5-FU. 12/42 percutaneous and 14/58 surgical had previously had the other technique
Sameshima 2007 [52]	Japan	Prospective single-arm cohort; HAI 1999–2005	Femoral; side-hole, tip fixed in GDA with coils; port	42	After resection of primary; 5-FU
Hildebrandt 2007 [53]	Germany	Prospective phase II with historical surgical comparison	Access artery not specified; port sited inferior to the groin (*n* = 41) vs. surgical (*n* = 40)	41 (+40 surgical)	Liver-only CRLM; 5-FU-based
Iguchi 2008 [57]	Japan	Retrospective single-centre cohort, 2000–2004	Left subclavian; side-hole, tip fixed in GDA with coils + NBCA/lipiodol; port	21	Synchronous unresectable with liver dysfunction; preoperative HAI 5-FU before resection of the primary colorectal tumour
Seki 2008 [58]	Japan	Retrospective single-arm cohort, four technique subgroups, 1995–2005	Surgically exposed left axillary branch (*n* = 122) or inferior epigastric artery (*n* = 13); conventional end-hole (*n* = 10) vs. fixed-tip (*n* = 77), modified fixed-tip (*n* = 24), long tapered (*n* = 24); port	135	Unresectable; 5-FU
Nishiofuku 2010 [59]	Japan (four centres)	Retrospective multicentre cohort, 2005–2008	Left subclavian (*n* = 41) or right femoral (*n* = 14); side-hole, tip fixed in GDA; port	55	Refractory to oxaliplatin, irinotecan and 5-FU
Deschamps 2011 [34]	France	Retrospective comparative cohort, single centre, 2004–2008	Femoral; side-hole, tip fixed in GDA with coils; port (*n* = 67) vs. surgical (*n* = 59)	67 (+59 surgical)	Mixed unresectable and adjuvant; oxaliplatin with systemic 5-FU/LV
Lee 2011 [60]	Korea	Retrospective single-centre cohort, 2004–2009	Right femoral; side-hole, tip fixed in GDA with coils; port in inner thigh	14	Refractory after systemic 5-FU; 5-FU/LV
Arai 2012 [54]	Japan (five centres)	Prospective multicentre phase I/II trial, 2003–2008	Left subclavian in all; port; embolisation of aberrant and collateral vessels	25	Unresectable liver-limited; HAI 5-FU with systemic irinotecan
Shi 2015 [61]	China	Retrospective analysis of a prospective database, 2007–2012	Right femoral; side-hole, tip in GDA (*n* = 45) or peripheral hepatic branch (*n* = 9); port in inner thigh	54	Unresectable synchronous liver-only, primary in situ; FUDR with systemic XELOX, conversion intent
Arai 2015 [55]	Japan (nine centres)	Prospective multi-institutional phase II trial (JFMC28), 2000–2002	Subclavian, standard IR technique; port; heparinised catheter in 75%	77	Primary resected, liver metastases untreated; weekly 5-FU
Goi 2015 [62]	Japan	Single-centre case series, 2011–2014	Right femoral; side-hole, tip in GDA, coil embolisation; device type not stated	10	Chemotherapy-refractory; 5-FU/LV. No device outcome reported
Kusano 2017 [51]	Japan (multicentre)	Randomised phase III trial (JFMC29-0003), 2000–2003; HAI arm eligible	Route not specified; placed by an experienced radiologist, embolisation of aberrant vessels; implanted reservoir + external pump	44 (HAI)	Adjuvant after curative resection, vs. systemic 5-FU
Sato 2018 [56]	Japan (two centres)	Prospective phase I/II trial, 2010–2012	Route not specified; side-hole with distal fixation; port	13	Refractory; HAI oxaliplatin with IV 5-FU/LV
Kedra 2023 [63]	France	Retrospective single-centre cohort, 2021–2022	Ultrasound-guided femoral; modified 5F catheter, hand-punched side hole, tip in GDA (*n* = 5) or peripheral branch (*n* = 10); paediatric port	14 (15 procedures)	Liver-dominant unresectable; oxaliplatin or 5-FU, using a device workaround during the catheter shortage

Abbreviations: 5-FU, fluorouracil; CRC, colorectal cancer; CRLM, colorectal liver metastases; FUDR, floxuridine; GDA, gastroduodenal artery; HAI, hepatic arterial infusion; IR, interventional radiology; *n*, number of patients; RCT, randomised controlled trial.

Two reports identified by citation searching could not be obtained in full text [32,64], and seven of the 22 reports assessed were excluded (Figure 1). Four described percutaneous port implantation in detail but in populations only 71–95% colorectal-primary with no extractable subgroup [65,66,67,68]; two used both techniques within one population without stratified reporting [69,70]; and one was excluded as a cohort overlapping an included study [29], which was retained as the index report for its stricter success definition and single standardised catheter-tip technique [34].

Access route and device type were not uniform (Table 1) and both are material sources of clinical heterogeneity. Three studies warrant comment. In Seki 2008 [58] the access vessel was exposed by surgical cutdown in all 135 patients, even though catheter placement and port implantation were radiological; it is therefore excluded from the primary analyses and added in a sensitivity analysis for every device outcome (Table 2). Oberfield 2004 [40] used a transbrachial catheter attached to an external portable pump rather than an implanted port, a configuration no longer in use, and it contributes to no pooled analysis. Goi 2015 [62] never states whether its device was a port and reports no insertion outcome, so it contributes to none of the pooled analyses.

**Table 2:** Random-effects proportion meta-analyses of percutaneous device outcomes.

Analysis	k	Events/N	Pooled % (FT, Random Effects)	95% CI	I^2^ (%)	GLMM % (95% CI)
Technical success, primary: fully percutaneous implanted ports [34,52,53,54,56,57,59,63]	8	276/278	99.9	98.7–100.0	0.0	99.6 (88.6–100.0)
Sensitivity: adding Seki 2008 [58] (surgically exposed access artery)	9	411/413	100.0	99.4–100.0	0.0	99.9 (85.0–100.0)
Subgroup: prospective	4	121/121	100.0	98.5–100.0	0.0	NE
Subgroup: retrospective	4	155/157	99.6	97.2–100.0	0.0	98.7 (95.1–99.7)
Subgroup: enrolment began before 2005	5	194/196	99.6	97.6–100.0	0.0	99.1 (90.2–99.9)
Subgroup: enrolment began 2005 or later	3	82/82	100.0	98.3–100.0	0.0	NE
Device-attributable definitive failure, primary [34,52,53,55,57,59,60,61,63]	9	39/374	6.9	2.1–13.7	74.5	6.9 (3.0–15.2)
Sensitivity: adding Seki 2008 [58], all arms	10	69/509	8.4	3.2–15.4	79.7	8.2 (3.9–16.6)
Sensitivity: adding Seki 2008 [58], side-hole arms only (22/125)	10	61/499	8.1	3.3–14.4	75.8	8.2 (4.1–16.0)
Sensitivity: excluding Iguchi 2008 [57] (HAI stopped before resection of the primary colorectal tumour)	8	39/353	8.1	2.8–15.5	74.6	8.3 (4.0–16.4)
Sensitivity: adding Sato 2018 [56] (0/13; no device abnormality reported, reasons for stopping not reported)	10	39/387	6.2	1.7–12.4	72.7	6.1 (2.5–14.3)
Sensitivity: adding Arai 2012 [54] (1/25 never began HAI after placement-related cerebral infarction)	10	40/399	6.7	2.2–12.8	71.7	6.6 (3.0–13.9)
Sensitivity: Lee 2011 [60] counted as 2/14 (displacement also reported as ending HAI)	9	40/374	7.4	2.4–14.4	75.0	7.5 (3.4–15.7)
Sensitivity: studies at low or moderate overall risk of bias [34,52,53,55,59,63]	6	38/285	11.6	6.3–18.0	51.6	12.6 (8.1–19.0)
Catheter dislodgement or tip migration, primary [34,52,53,56,57,60,61,63]	8	21/255	5.8	1.6–11.6	53.6	6.0 (2.5–13.4)
Sensitivity: adding Seki 2008 [58]	9	32/390	6.2	2.7–10.8	47.1	6.8 (3.6–12.7)
Sensitivity: excluding counts derived from revision data [34,53]	6	5/158	2.1	0.1–5.7	0.0	3.2 (1.3–7.4)
Subgroup: port checked angiographically before every course or every four weeks [34,53,63]	3	17/111	14.8	8.4–22.4	0.0	15.3 (9.7–23.3)
Subgroup: less frequent or unspecified device surveillance [52,56,57,60,61]	5	4/144	1.8	0.0–5.5	0.0	2.8 (1.0–7.2)
Hepatic arterial occlusion or thrombosis, primary [34,52,57,59]	4	7/174	3.4	0.9–7.0	0.0	4.0 (1.9–8.2)
Sensitivity: adding Seki 2008 [58], all arms	5	32/309	5.8	0.8–13.9	79.7	5.4 (1.9–14.5)
Sensitivity: adding Seki 2008 [58], side-hole arms only (20/125)	5	27/299	5.5	1.0–12.4	73.0	5.4 (2.0–13.6)
Sensitivity: adding Hildebrandt 2007 [53] event count (seven thromboses)	5	14/215	5.2	1.1–11.3	58.3	5.3 (2.1–12.8)

Pooled proportions were estimated using a random-effects model with Freeman–Tukey (FT) double arcsine transformation and the DerSimonian–Laird estimator, back-transformed using the harmonic mean sample size, and with a binomial–normal random-intercept logistic generalised linear mixed model (GLMM) fitted by maximum likelihood. All counts are patients, except the sensitivity analysis adding Hildebrandt 2007 to hepatic arterial occlusion, which uses an event count because the number of patients affected could not be determined. Primary analyses include only fully percutaneous implanted ports. NE, not estimable (every study at 100%). Crude proportions with exact 95% CIs for every analysis are given in Supplementary Table S2b. Between-subgroup test for device surveillance: Q = 12.22, df = 1, *p* < 0.001. Overall risk of bias as in Supplementary Table S1a. Abbreviations: CI, confidence interval; I^2^, proportion of total variability attributable to between-study heterogeneity; k, number of studies.

### 3.2. Risk of Bias

Judged for the device outcomes used here, overall risk of bias was low in one study [63], moderate in six [34,52,53,55,58,59] and high in eight [40,51,54,56,57,60,61,62]; item-level judgements and their basis are given in Supplementary Table S1a. Outcome ascertainment most separated the studies. Four checked the port by angiography or CT angiography before every course or at least four-weekly [34,53,54,63], five at scheduled intervals of one to three months or every ten infusions [40,52,55,58,59] and the remainder only at implantation, at two fixed time points or when a problem was suspected [51,56,57,60,61,62]; only one study graded device complications [63], and four stated device outcomes as an objective [34,53,58,63]. For patient selection, three cohorts were selected subsets of larger institutional or trial populations [53,57,61] and two were defined by receipt of HAI rather than by implantation, so that patients implanted but never treated cannot be seen [59,60]. For completeness, Deschamps 2011 did not report device outcomes for nine successfully implanted percutaneous patients who received HAI elsewhere [34], and the randomised trial reported device data for 37 of its 44 analysed patients [51]. On RoB 2 the randomised trial [51] had low risk of bias for its central, stratified randomisation but raised some concerns overall for the device outcomes used here (Supplementary Table S1b): it was open-label, closed after enrolling 91 of a planned 300 patients and gave catheter-related complications as an approximate proportion without a prespecified definition. The studies with the least intensive surveillance tended to report the fewest device failures (Table 3); this observation is exploratory, since differences between studies cannot show whether ascertainment raised or lowered the pooled device estimates, or by how much (Section 3.4).

### 3.3. Technical Success

Eight studies of fully percutaneous implanted ports reported technical success explicitly [34,52,53,54,56,57,59,63], and in seven of these implantation succeeded in every patient (Table 3); Seki 2008 [58] also reported success in all 135 patients. Deschamps 2011 [34] is the only study reporting primary failures, implantation having succeeded in 65 of 67 patients (97%), both failures being attributable to coeliac trunk stenosis; it also applied the strictest success criterion, requiring complete hepatic opacification without extrahepatic perfusion rather than catheter placement alone. Across the eight studies, implantation succeeded in 276 of 278 patients, a crude rate of 99.3% (exact 95% CI 97.4–99.9%); Kedra 2023 [63] reported success per procedure (15 of 15), and its patient-level count (14 of 14) was used. Six studies did not state a success rate [40,51,55,60,61,62] and contribute nothing to this outcome, since success was not inferred from the absence of reported failures.

The pooled Freeman–Tukey estimate was 99.9% (95% CI 98.7–100.0%), with no detectable heterogeneity (I^2^ = 0.0%; Q = 2.66, df = 7, *p* = 0.91), and the GLMM gave 99.6% (95% CI 88.6–100.0%), its wider interval reflecting how little information proportions at the boundary carry. This should not be read as a claim that implantation never fails: two failures did occur, and the Freeman–Tukey transformation behaves unstably when proportions sit at or near the 100% boundary, which seven of the eight do. The crude proportion and its exact interval are therefore the more informative summary: they show that primary implantation failure was uncommon in these series, compatible with a failure rate of up to about 2.6%, but two events cannot establish that rate, and implantation failure may be inconsistently reported by small single-centre series. This is the principal reason technical success is not treated as the primary outcome here. Adding Seki 2008 [58] did not change the estimate, and subgroup analyses showed no difference by design (Q = 0.52, *p* = 0.47) or treatment era (Q = 0.26, *p* = 0.61; Table 2). For context, two of the comparative studies reported surgical implantation success of 38 of 40 (95%) [53] and 58 of 59 (98%) [34]; these arms were not pooled, since three small non-randomised studies spanning four decades of technique evolution, one using a historical control and none adjusting for confounding by indication, cannot support a comparative conclusion.

### 3.4. Device-Attributable Definitive Failure

Nine studies of fully percutaneous implanted ports reported explicitly how many patients permanently discontinued HAI because of a device or catheter problem [34,52,53,55,57,59,60,61,63]. Rates ranged from 0% to 21.4% and are given per study in Table 3, with a pooled estimate of 6.9% (95% CI 2.1–13.7%; 39/374) and substantial heterogeneity (I^2^ = 74.5%; Q = 31.35, df = 8, *p* < 0.001); the GLMM gave 6.9% (95% CI 3.0–15.2%). The crude proportion was higher, 39 of 374 patients (10.4%, exact 95% CI 7.5–14.0%), because the random-effects model gives relatively more weight to the three studies reporting no failure [57,61,63], whereas the crude proportion is dominated by the larger cohorts reporting 15–21% [34,53,55]. Restricted to the six studies at low or moderate overall risk of bias, all of which monitored the port at scheduled intervals, the estimate was 11.6% (95% CI 6.3–18.0%; 38/285; I^2^ = 51.6%; GLMM 12.6%, 8.1–19.0%); the three studies at high risk in this analysis reported one failure among 89 patients and had the least device surveillance [57,60,61]. Sato 2018 [56], which reported no reasons for stopping HAI but stated in its discussion that no tip migration or perfusion abnormality occurred, was moved from the primary analysis to a sensitivity analysis so that no zero count rests on inference; adding it gave 6.2% (95% CI 1.7–12.4%). Adding Seki 2008 [58], which reported the highest rate (22.2%), raised the estimate to 8.4% (Table 2). These are crude proportions over follow-up that differed widely between studies, not cumulative probabilities of device failure. Duration of device exposure, catheter-tip technique (Section 3.6) and intensity of device surveillance are possible contributors to the heterogeneity, but none could be tested formally and all changed alongside treatment era, regimen and patient selection. Device exposure, observation period and the reasons for stopping HAI are tabulated by study in Table 4. Exposure ranged from a mean of 4.5 HAI courses in Iguchi 2008 [57], in which HAI was stopped one week before resection of the primary colorectal tumour in the 19 patients who underwent it (mean 3.9 courses beforehand), after which 16 received systemic chemotherapy with or instead of HAI and the number continuing HAI was not reported, to a mean follow-up of 569 days in Seki 2008 [58]. The association is not uniform: Shi 2015 [61], which also reported no definitive failure, gave a median of nine HAI courses in resected patients, most of whom resumed HAI after conversion surgery, but described no scheduled surveillance of the device. Where reasons for stopping HAI were reported, disease progression was the most frequent, and device problems accounted for 0–22% of patients (Table 4). In Deschamps 2011 [34], technical success is reported for all 67 attempted implantations, whereas device outcomes are reported for the 56 percutaneous patients treated at the authors’ institution; twelve patients across both arms, nine of them percutaneous by subtraction, received HAI elsewhere and were not analysed, and no device failure between implantation and the first course was reported.

Three further studies did not contribute to the primary estimate. The randomised trial [51] used an implanted reservoir connected to an external pump, placed by a route not specified, and so is not a fully percutaneous implanted port; it reported technical discontinuation in one of 37 patients with data, but also cumulative hepatic arterial catheterisation failure of 14.8% at three months and 16.7% at six months and approximately 20% of patients developing catheter-related complications, without numerators. Oberfield 2004 [40] reported no composite device-failure endpoint. Arai 2012 [54] reported device performance only during its five-cycle protocol and did not record post-protocol treatment; one of its 25 patients never began HAI after a cerebral infarction attributed to catheter placement, and counting this patient as a definitive failure changed the pooled estimate little (6.7%; Table 2). Lee 2011 [60] lists catheter thrombosis as the only device-related reason for stopping HAI but elsewhere describes a displacement that led to catheter removal and discontinuation. Because its tabulated reasons for stopping HAI account for all 14 patients, the patient with displacement is already counted under another reason and cannot be identified, so the primary analysis uses the tabulated count; counting both gave 7.4% (Table 2).

### 3.5. Catheter Dislodgement and Tip Migration

Catheter dislodgement or tip migration is plausibly attributable to the absence of a sutured arterial anchor and is therefore the mechanistically specific failure mode of percutaneous placement. Eight fully percutaneous implanted-port studies reported the number of patients affected [34,52,53,56,57,60,61,63], with rates from 0% to 19.5% (Table 3) and a pooled estimate of 5.8% (95% CI 1.6–11.6%; 21/255; GLMM 6.0%, 2.5–13.4%) with moderate heterogeneity (I^2^ = 53.6%; Q = 15.10, df = 7, *p* = 0.04). In two of these the source lists migration as events, but the number of patients affected is fixed by the reasons given for revision. In Deschamps 2011 [34] the revision reasons for the 33 revised patients sum exactly to 33 (extrahepatic perfusion 19, incomplete hepatic perfusion 5, catheter occlusion 1, migration 8), and in Hildebrandt 2007 [53] the reasons for first revision in the 23 revised patients sum exactly to 23 (dislocation 8, leakage 5, thrombus or embolism 5, disconnection 3, occlusion 1, infection 1); since each study also reports exactly eight migration events, eight distinct patients were affected in each (Supplementary Table S2a). These are two of the three studies that checked the port angiographically before every course or every four weeks, and dislodgement was markedly more frequent in that group (14.8%, 95% CI 8.4–22.4%; 17/111) than in studies with less frequent or unspecified surveillance (1.8%, 95% CI 0.0–5.5%; 4/144; between-group Q = 12.22, *p* < 0.001), a post hoc, exploratory comparison confounded by era, device and centre. Excluding the two studies gave 2.1% (95% CI 0.1–5.7%), but because they are among the studies most likely to detect migration, that figure may underestimate dislodgement and is not a correction; adding Seki 2008 [58] gave 6.2% (Table 2). Oberfield 2004 [40] is reported separately because its percutaneous arm used an external portable pump rather than an implanted port; displacement occurred in 25 of 42 patients (60%), against eight of 58 (14%) in its surgical arm (*p* < 0.001), an order of magnitude above every implanted-port series here and best understood as a property of an obsolete device configuration rather than of percutaneous access. Displacement was usually correctable without surgery: no catheter-tip dislocation or catheter–port disconnection resulted in terminal device failure in one series [53], and all seven catheter dysfunctions were treated successfully in another [63].

### 3.6. Hepatic Arterial Occlusion and Thrombosis

Four fully percutaneous studies reported the number of patients with hepatic arterial occlusion or thrombosis [34,52,57,59], with rates from 0% to 7.3% (Table 3) and a pooled estimate of 3.4% (95% CI 0.9–7.0%; 7/174; GLMM 4.0%, 1.9–8.2%) without detectable heterogeneity (I^2^ = 0.0%). Hildebrandt 2007 [53] reported seven total thromboses as events among 39 complications in 26 patients, so the number of patients affected is unknown, and Seki 2008 [58] used surgical access; adding them gave 5.2% and 5.8% respectively, the latter with substantial heterogeneity (I^2^ = 79.7%; Table 2). Two further studies state that no catheter occlusion [61] and no perfusion abnormality [56] occurred; hepatic arterial occlusion was not inferred from these statements. The single events described as catheter thrombosis in two studies [60,63] are not hepatic arterial occlusion and were not counted.

Catheter-tip technique may contribute to variation in this outcome. Seki 2008 [58] is the only included study reporting outcomes separately by technique within one institution. Hepatic arterial occlusion occurred in five of 10 patients (50%) treated by the conventional unfixed end-hole method against 20 of 125 (16%) treated by one of three side-hole or fixed-catheter-tip methods (*p* = 0.0106 in the source study), with one-year hepatic arterial patency of 38.9% against 76.3–95.0% (*p* ≤ 0.0014 for each) and median time to treatment discontinuation of 3.2 against 11.7–14.4 months. The four techniques were introduced sequentially over 1995–2005, so technique is confounded by era within that series, and restricting it to its side-hole arms still left substantial heterogeneity (5.5%; I^2^ = 73.0%; Table 2); technique is therefore a plausible but unproven contributor. Six studies fixed the tip in the gastroduodenal artery by coils, with or without n-butyl cyanoacrylate, in every patient [34,52,57,59,60,62] and one in most [61]; among the four of these reporting hepatic arterial occlusion explicitly, the rate was 0–7.3%.

### 3.7. Complications, Revision and Device Durability

Composite complication definitions and granularity varied substantially (per-patient versus per-catheter versus per-treatment-course), precluding pooling of an any-complication endpoint; individual findings are in Table 3 and rates ranged from no device complication of any kind [57] to 63% [53], where treatment-limiting vascular complications nonetheless affected only 12.2%, against 37.5% in the surgical arm. Deschamps 2011 [34] found no difference in the per-course complication rate between techniques (*p* = 0.90) but a distribution differing in a pattern consistent with the mechanics of each: extrahepatic perfusion (*p* = 0.04) and catheter-tip migration (*p* = 0.005) were more frequent after percutaneous placement, whereas catheter occlusion unresponsive to fibrinolysis (*p* = 0.006) and hepatic artery dissection or thrombosis (*p* = 0.004) were more frequent after surgical placement. The largest prospective multi-institutional trial reported a 19% catheter-related complication rate across nine institutions, with salvage in 70% of complications arising within three months and 30% of later ones [55]. The mechanisms of these complications and the measures used to prevent and manage them are summarised in Table 5.

The two comparative studies report device performance over time in detail (Table 6), and both show more frequent but more easily correctable revisions translating into good device survival. Hildebrandt 2007 [53] required revision in 23 of 41 percutaneous patients (56%), all managed endovascularly, yet secondary device failure occurred in only 17% and 77% of devices remained functional at 12 months, against 50% secondary failure in the surgical arm. Deschamps 2011 [34] found primary functionality identical between techniques (4.80 versus 4.82 courses, *p* = 0.99) but functionality after revision significantly greater for percutaneous devices (9.18 versus 5.95 courses, *p* = 0.004), a difference consistent with, though not shown to result from, the surgical catheter being sutured within the gastroduodenal artery and therefore not exchangeable. Reintervention was likewise common but effective in the newer series [52,59,63]. Reintervention was nonetheless a substantial burden: in the two series reporting it per patient, 56% [53] and 59% [34] of percutaneous patients needed at least one revision, against 10% and 24% after surgical placement.

### 3.8. Oncological and Survival Outcomes

Oncological outcomes are exploratory and reflect chemotherapy regimen, line of therapy and patient selection at least as much as insertion technique; they are summarised in Table 7 and should be read in that light. The studies span first-line, salvage and adjuvant settings, and response rates ranged accordingly from 7% in a heavily pretreated salvage cohort [60] to 100% hepatic response in a first-line conversion cohort with the primary in situ [61], with the shortest survival in salvage cohorts and the longest in less heavily pretreated populations. Oberfield 2004 [40] found no significant difference between its arms in response (48% versus 34%, *p* = 0.22) or median survival (10.6 versus 13.0 months, *p* = 0.39). In Seki 2008 [58], median overall survival was 21.1–23.1 months across the side-hole methods against 13.1 months for the conventional method, but the techniques were used sequentially over 1995–2005, the conventional group comprised ten patients and the difference is confounded by era, systemic therapy and patient selection; it should not be attributed to catheter technique. The randomised trial [51], in the adjuvant setting, found no significant difference in five-year overall survival between its arms (34.9% versus 59.0%, *p* = 0.146) but significantly lower five-year relapse-free survival in the hepatic arterial arm (20.4% versus 44.1%, *p* = 0.038), in an underpowered trial.

## 4. Discussion

This review identified fifteen studies of percutaneous or image-guided HAI port insertion for colorectal liver metastases, contributing 654 percutaneously treated patients and spanning more than five decades of technique evolution. Primary implantation rarely failed, whereas device-related complications and reintervention were common; many were corrected endovascularly, but only one study graded severity, so the data do not show that complications were predominantly minor. Technical success was achieved in 276 of 278 patients across the eight fully percutaneous studies reporting it explicitly, with no detectable heterogeneity, and both failures were anatomical rather than technical, arising from coeliac trunk stenosis in the study applying the strictest success definition in the literature. Primary implantation failure was therefore uncommon in these series, but two events cannot establish its rate (exact 95% CI for failure 0.1–2.6%), and small series may under-report it. The clinically decisive question is what happens to the device afterwards.

On that question the evidence is less secure. Device-attributable definitive failure was estimated at 6.9% by random-effects pooling across nine studies, against a crude 10.4% (39 of 374 patients) and 11.6% in the six studies at low or moderate risk of bias. These derive from different analysis sets and do not define a single risk interval; all three are proportions over heterogeneous and often short observation periods rather than the probability that a device ultimately fails, and the lowest is the least conservative. Catheter dislodgement or tip migration, the failure mode most specific to a catheter not sutured to the arterial wall, occurred in a pooled 5.8%, rising to 14.8% where the port was checked angiographically before every course or every four weeks, and was usually correctable endovascularly; patient-level hepatic arterial occlusion, which often ends treatment, was reported by only four studies (3.4%). Revision, by contrast, was needed in more than half of percutaneous patients in both comparative series that reported it [34,53]. These describe a device that is easy to place but carries a substantial reintervention burden, which should be weighed alongside its technical feasibility.

The substantial heterogeneity around definitive failure (I^2^ = 75%) is not explained by this analysis. Duration of device exposure is a possible contributor: the study in which HAI was given for a mean of 3.9 courses before resection of the primary colorectal tumour reported no device failures [57], whereas cohorts treated for longer reported up to one in five, so the pooled proportion is likely to understate failure in patients treated to progression. Catheter-tip technique is another. Intensity of device surveillance is a third: the studies that checked the port least often reported the fewest failures, which may reflect ascertainment, although differences between studies cannot show whether this raised or lowered the estimates, or by how much. None could be tested by meta-regression in so few small studies, and all are confounded by treatment era, regimen and patient selection, which changed in parallel. In the only within-study comparison, which was observational and sequential, hepatic arterial occlusion occurred in five of 10 patients treated by the conventional unfixed end-hole method against 20 of 125 treated by side-hole or fixed-tip methods within a single institution (*p* = 0.0106), with one-year patency of 38.9% against 76.3–95.0% [58]. This is mechanistically plausible: a catheter unanchored at the point of arterial entry is intrinsically more prone to migration, but such complications are directly visualised and often correctable under fluoroscopy without further surgery. The technique literature developed specifically to address that vulnerability, from the conventional end-hole approach described in 1999 [74] through side-hole placement with distal fixation formalised in 2003 [71] and refined thereafter with coil and n-butyl cyanoacrylate embolisation around the catheter tip [72,73,75], so earlier cohorts, including one using a 1976–1995 technique [40], may understate what contemporary technique achieves. Extrahepatic perfusion is not unique to percutaneous placement in any case: even with routine surgical skeletonisation, 7.3% of patients in one series of 327 pumps required arteriography and embolisation for it [76].

The three studies with a surgical comparator arm were deliberately not pooled, but what they show consistently is a difference in the character rather than the frequency of device problems (Table 3 and Table 6): percutaneous devices fail more often by migration and extrahepatic perfusion, surgical devices by arterial dissection, thrombosis and irreversible occlusion and the percutaneous device is the one that can more readily be exchanged. Deschamps 2011 [34] makes this concrete, reporting identical primary functionality between techniques but significantly greater functionality after revision for percutaneous devices (9.18 versus 5.95 courses, *p* = 0.004), which may reflect, but was not shown to result from, the fact that the surgically sutured catheter cannot be removed. Two studies temper an otherwise favourable account. Arai 2015 [55], the largest prospective multi-institutional trial here, reported a 19% catheter-related complication rate across nine institutions and concluded that percutaneous placement had not reduced complications relative to surgical series; its authors suggested that variable operator expertise across the participating institutions may have contributed, but this cannot be separated from differences in era, regimen, patient selection and the intensity with which complications were sought, and the lower rates in single-centre series may also reflect selective publication by experienced centres. The randomised trial [51] closed early with fewer than a third of its planned sample, the investigators attributing poor accrual mainly to the invasiveness and technical difficulty of catheter placement. Feasibility of an individual procedure is not the same as feasibility of a programme.

A further factor, absent from the included studies but relevant to their contemporary applicability, is the disruption to HAI device supply over the past decade. The Codman 3000, the principal dedicated implantable pump in North America, was discontinued in 2018 [30], leaving no device approved for this indication and prompting centres to improvise hybrid catheter and pump combinations under investigational device exemptions until a successor was approved [31]. The percutaneous side has not recovered in the same way: the manufacturer of a widely used dedicated percutaneous arterial port catheter withdrew it under new medical device regulations, and the most recent study included here exists because of that shortage, describing a modified diagnostic catheter with a hand-punched side hole coupled to a paediatric port [63]. Device availability may therefore now cut against percutaneous placement, and future studies should account for it.

With increasing availability of devices and expertise for image-guided HAI port placement, HAI may be feasible in selected patients, particularly where access to surgical pump placement or radioembolisation is limited; a durably functioning arterial port is also the platform for other intra-arterial strategies in treatment-refractory disease [77]. The advantage of the percutaneous route is not simply that it avoids surgery, since surgical pump placement is usually performed concurrently with another indicated procedure rather than as a standalone operation [27]—with resection of the primary in synchronous disease, with staged hepatectomy in resectable bilobar disease and with hepatectomy in limited recurrent disease—so a standalone operation for pump placement is largely confined to metachronous, unresectable disease. International guidelines recognise SIRT, stereotactic body radiotherapy (SBRT) and HAI as liver-directed options for CRLM, but none addresses the route of arterial access. That gap is relevant to access, because NCCN guidance restricts HAI eligibility to candidates for major surgery [4], a criterion framed around surgical pump placement. Feasible percutaneous access does not, however, make a patient suitable for HAI chemotherapy: performance status, hepatic reserve, extrahepatic disease and tolerance of regional and systemic toxicity still determine eligibility, and no included study tested whether patients unfit for surgery benefit. These data support percutaneous placement as a route of access for patients already judged suitable for HAI, not wider eligibility for HAI itself. Retrospective comparisons favour HAI over yttrium-90 transarterial radioembolisation (TARE): in a propensity-matched six-institution analysis, TARE carried a higher risk of all-cause mortality than HAI after first-line (HR 1.46, 95% CI 1.02–2.08) and second-line (HR 1.96, 95% CI 1.32–2.89) chemotherapy, with more frequent conversion to resection after HAI, though with no difference in the refractory setting [78]; a single-institution comparison in pretreated disease reported a similar advantage (median overall survival 31.2 versus 16.3 months) [79]. SIRT nonetheless has stronger prospective support: the phase III EPOCH trial (*n* = 428) showed improved progression-free survival (HR 0.69, 95% CI 0.54–0.88) and objective response (34.0% versus 21.1%) when TARE was added to second-line chemotherapy, though without an overall survival benefit (14.0 versus 14.4 months) [80]. Reflecting that difference, the 2022 ASCO guideline addresses SBRT and SIRT as liver-directed options, recommending SBRT and advising against routine SIRT, while not addressing HAI at all [5]. It may be hypothesised that image-guided HAI could achieve outcomes comparable to SIRT, but this has not been tested prospectively.

Thermal ablation and transarterial chemoembolisation address different clinical problems. Ablation treats a limited number of small metastases with curative intent: added to systemic therapy it prolonged overall survival in the randomised phase II CLOCC trial (HR 0.58, 95% CI 0.38–0.88) [81], and in COLLISION it was non-inferior to resection for metastases of 3 cm or less, with fewer adverse events (19% versus 46%) [82]. HAI, like radioembolisation and irinotecan-eluting bead chemoembolisation, which prolonged median overall survival over FOLFIRI (22 versus 15 months) in a small phase III trial [83], treats the whole arterial territory of the liver and is directed at multifocal or bilobar disease beyond the reach of ablation. Unlike these, it depends on a device that must remain functional for months, so device durability is a consideration specific to HAI. No randomised trial has compared HAI with ablation or chemoembolisation, and the modalities are better regarded as complementary than competing.

HAI is also used outside colorectal disease, although not necessarily with the device studied here. In unresectable intrahepatic cholangiocarcinoma, HAI floxuridine delivered through surgically implanted pumps, combined with systemic gemcitabine and oxaliplatin, achieved a median overall survival of 25.0 months [84], and arterial oxaliplatin and fluorouracil improved overall survival over sorafenib in advanced hepatocellular carcinoma in the phase III FOHAIC-1 trial (13.9 versus 8.2 months) [85]. These populations differ in device, liver function, arterial anatomy and treatment duration, and the device estimates here should not be extrapolated to them.

Hepatic arterial injury from HAI also has consequences beyond treatment itself. Arterial thrombosis was the leading treatment-limiting device complication in one included series [53], arterial dissection or thrombosis was more frequent after surgical than percutaneous placement in another [34] and floxuridine-related biliary sclerosis reached 6% at five years among 2239 patients treated at one centre [86]. This matters because liver transplantation is now an option for selected patients with permanently unresectable CRLM, having improved five-year overall survival from 12.6% to 56.6% in the randomised TransMet trial [87]. Previous HAI pump therapy has limited access to transplantation [88], and whether the pump is a technical obstacle to the arterial anastomosis has been debated [89], although in a six-centre series of 35 deceased-donor transplants for CRLM, prior pump therapy in 19 patients was not associated with differences in disease-free or overall survival [88]. Whether percutaneous placement, which avoids hilar dissection, affects the recipient artery differently from surgical placement has not been studied, and arterial occlusion, dissection or stenosis should be documented in any patient who may later be considered for transplantation.

Several limitations apply. Fourteen of the fifteen studies are non-randomised and nine retrospective, with the attendant risk of selection and reporting bias and confounding by treatment era, and the single randomised trial raises some concerns on RoB 2 for the device outcomes used here. Judged for the device outcomes themselves, eight studies were at high risk of bias, most often because the device was not monitored at fixed short intervals, and the studies that did monitor it closely reported more device problems, although this exploratory between-study observation cannot establish the direction or size of any ascertainment bias. Nine were conducted in Japan, where side-hole placement with distal fixation has dominated since the early 2000s, so the durability estimates are weighted towards one national technical school. One included study [34] was identified only through citation searching because it used different terminology for HAI, and other eligible studies described in non-standard terms may have been missed. Several studies reported complications as events rather than affected patients; such counts were used only where the number of patients affected could be determined, which left the patient-level occlusion estimate resting on four small studies [34,52,57,59]. Complication reporting remains heterogeneous in denominator and severity, with only one study applying a formal grading system, which is why an any-complication endpoint could not be pooled. Restricting inclusion to exclusively colorectal cohorts guards against contamination but excluded several large series that were 71–95% colorectal [65,66,67,68]. Finally, enrolment spans 1970 to 2022 and most pooled patients were treated before 2010, so interpretation risks conflating technique with era. Restriction of the primary analyses to fully percutaneous implanted ports, the patient-level denominator rule and the GLMM were introduced at peer review rather than prespecified, as were the domain-based risk-of-bias assessment and the analyses by risk of bias and device surveillance. The main estimates were consistent between the Freeman–Tukey and GLMM models, but the definitive-failure estimate rose substantially, from 6.9% to 11.6%, when restricted to studies at lower risk of bias, so the primary figure may underestimate device failure; the two estimates derive from different analysis sets and are reported separately. Prospective multicentre studies using a common device-outcome definition set, reporting device failure as a time-to-event outcome rather than a crude proportion, are needed before these estimates can be regarded as practice-defining.

## 5. Conclusions

In the series available, percutaneous, image-guided insertion of hepatic arterial infusion ports for colorectal liver metastases rarely failed technically, although two failures among 278 patients cannot define the failure rate, and implantation success should continue to be reported in a standardised way. Device durability and reintervention are the less certain questions: permanent loss of HAI to a device problem within the follow-up reported was estimated at 6.9% in the primary analysis and 11.6% in studies at lower risk of bias, estimates from different analysis sets rather than a single risk range; revision was frequent; and studies that monitored the device more closely reported more problems. Catheter-tip fixation technique, duration of follow-up and intensity of surveillance are possible but unproven contributors to the variation, and these predominantly observational data establish the feasibility of percutaneous access rather than patient suitability for HAI chemotherapy.

## Figures and Tables

**Table 3:** Technical success, device-attributable failure and device-related complications by study.

Study	Technique	*n*	Technical Success	Device-Attributable Definitive Failure	Device-Related Complications
Oberfield 2004 [40]	Percutaneous (external pump) vs. surgical	42 vs. 58	NR	NR	External-pump configuration. Thrombosis 36% vs. 33% (*p* = 0.83); emboli with ischaemia 10% vs. 0% *; displacement 60% vs. 14% *; catheter-site bleeding 26% vs. 0% *
Sameshima 2007 [52]	Percutaneous, fixed tip	42	42/42 (100%)	3/42 (7.1%)	Catheter failure 3/42 (occlusion 2, displacement 1); collateral supply re-embolised 3/42, HAI continued
Hildebrandt 2007 [53]	Percutaneous vs. surgical	41 vs. 40	41/41 (100%) vs. 38/40 (95%)	7/41 (17%) vs. 20/40 (50%) (*p* < 0.001) *	39 events in 26/41 (63%): tip dislocation 8, total thrombosis 7, partial thrombosis or embolism 6, leakage or extravasation 5, disconnection or rupture 5, dissection 3, port infection 3, reflux 1, other 1. Treatment-limiting vascular complications 12.2% vs. 37.5% *
Iguchi 2008 [57]	Percutaneous, fixed tip	21	21/21 (100%)	0/21	None reported
Seki 2008 [58]	Image-guided, four technique variants	135	135/135 (100%)	30/135 (22.2%)	Hepatic arterial occlusion 25, dislodgement 11, collateral supply 10, procedure-related 4, cholangitis 3, cerebral infarction 3, kinking 2, gastroduodenal ulcer 2, catheter occlusion 1
Nishiofuku 2010 [59]	Percutaneous, fixed tip	55	55/55 (100%)	4/55 (7.3%)	Collateral supply with drug maldistribution 7/55, all re-embolised and HAI continued; hepatic arterial occlusion 4/55
Deschamps 2011 [34]	Percutaneous vs. surgical	67 vs. 59	65/67 (97%) vs. 58/59 (98%)	12/56 (21%) vs. 19/55 (34%) (*p* = 0.12)	45 vs. 28 events (33/56 vs. 25/55 patients; per-course *p* = 0.90). Percutaneous: extrahepatic perfusion 20, tip migration 8, incomplete hepatic perfusion 6, access-site bleeding 3, port infection 2, catheter occlusion 1, arterial thrombosis 1. More frequent after percutaneous: extrahepatic perfusion, tip migration; after surgical: catheter occlusion, dissection or thrombosis (all *p* < 0.05) *
Lee 2011 [60]	Percutaneous, fixed tip	14	NR	1/14 (7.1%)	3/14 (21.4%): thrombosis 1, displacement 1 (device removed; also reported as ending HAI), catheter-related infection 1 (HAI continued)
Arai 2012 [54]	Percutaneous subclavian	25	25/25 (100%)	NR (1/25 never began HAI after placement-related cerebral infarction)	Cerebral infarction attributed to placement 1/25; HAI never initiated in this patient
Shi 2015 [61]	Percutaneous, fixed tip or peripheral	54	NR	0/54	6/54: displacement 2 (corrected radiologically, HAI continued), port-site infection needing debridement 4. No catheter occlusion
Arai 2015 [55]	Percutaneous subclavian, multi-institutional	77	NR	12/77 (15.6%)	Catheter-related complication rate 19%; early events mostly inadequate drug distribution (corrected angiographically), later ones occlusion or thrombosis. Salvage 70% early vs. 30% late
Goi 2015 [62]	Percutaneous femoral	10	NR	NR	No device or catheter complication reported
Kusano 2017 [51]	Radiologically placed, implanted reservoir (HAI arm of RCT)	44	NR	1/37 (2.7%) discontinued for a technical problem	Catheter-related complications in approximately 20%; none attributed to insertion
Sato 2018 [56]	Percutaneous, side-hole with distal fixation	13	13/13 (100%)	NR (no device abnormality reported; 0/13 in sensitivity analysis only)	No tip migration; no perfusion abnormality on DSA or CT angiography
Kedra 2023 [63]	Percutaneous femoral, modified catheter	14 (15 procedures)	14/14 patients (100%); 15/15 procedures; secondary (functional) success 14/15 (93.3%)	0/14	No procedural complication. Two Clavien–Dindo IIIa late complications; seven catheter dysfunctions (extrahepatic perfusion 2, disconnection 2, thrombosis 1, migration 1, incomplete perfusion 1), all corrected

Device complication columns are restricted to catheter- or device-related events; drug-related toxicity is excluded, since it reflects the chemotherapy regimen rather than the insertion technique. Deschamps 2011: technical success is given for all 67 attempted percutaneous implantations and device outcomes for the 56 percutaneous patients treated at the authors’ institution; nine successfully implanted patients received HAI elsewhere and were not analysed. Complication counts are events unless given as a proportion of patients. NR, not reported. * Statistically significant (*p* < 0.05) in the source study. Abbreviations: *n*, number of patients.

**Table 4:** Device exposure, observation period and reasons for stopping HAI by study.

Study	HAI Exposure	Observation Period	Implanted But Not in Device Outcomes	Reasons for Stopping HAI (Patients)
Oberfield 2004 [40]	Median 4 months of treatment (range 1–18)	Until death in all patients	NR	Not reported per patient; displacement interrupted treatment and required reinsertion
Sameshima 2007 [52]	Mean 36 infusions (range 10–98)	NR	None	Catheter failure 3 (occlusion 2, displacement 1); switched to systemic chemotherapy for extrahepatic metastases 22; other reasons NR
Hildebrandt 2007 [53]	260 courses in 41 patients; mean port duration 19 months	Mean 8.0 months	None	Disease progression 19; regular completion or ongoing 12; port complication 7; withdrawal 2; chemotherapy toxicity 1
Iguchi 2008 [57]	Mean 4.5 courses (range 1–15)	Median 309 days (range 51–998)	None	Stopped one week before resection of the primary colorectal tumour 19 (16 then received systemic chemotherapy with or instead of HAI; number continuing HAI NR); extrahepatic progression 2; device-related 0
Seki 2008 [58]	Median time to discontinuation 3.2–14.4 months by technique	Mean 569 days (range 50–2873)	None	Hepatic arterial occlusion 25; dislodgement 4; catheter occlusion 1; extrahepatic progression 33; no response 29; hepatic regrowth 14; complete response 8; refusal 2; death 1; not stated 18
Nishiofuku 2010 [59]	Median 12 courses (range 1–58) over a median 3.3 months	Median 6.1 months (range 0.5–27.5)	Not visible: cohort defined by receipt of HAI	Hepatic arterial occlusion 4; progression or death by data cut-off 53 (not mutually exclusive); continuing 2
Deschamps 2011 [34]	Mean 4.80 courses before and 9.18 after revision	Implantation to end of HAI	Nine of 65 implanted, treated elsewhere	Progression or death 32; port–catheter-related 12; response allowing curative treatment 5; chemotherapy toxicity 4; completion 3
Lee 2011 [60]	Mean 4.1 cycles (range 1–7)	Median 8.4 months (range 4.2–20)	Not visible: cohort defined by receipt of HAI	Disease progression 10; refusal 2; abdominal pain 1; catheter thrombosis 1 (a displacement ending HAI is also reported)
Arai 2012 [54]	Five protocol cycles planned; 106 protocol cycles delivered	Median 55.0 months (range 22.8–87.7)	Protocol HAI not started in three: leucocyte count 2 (HAI given off protocol), placement-related cerebral infarction 1 (HAI never given)	Five protocol cycles completed by most who started (13 of 14 in phase I); post-protocol HAI not recorded
Shi 2015 [61]	Median 4 cycles before resection (range 2–7); median 9 in total in resected patients	Median 40 months if resected, 18 months if not	None reported	Interrupted for conversion surgery 38 (HAI resumed after surgery in 34); gastroduodenal ulceration 1; device-related 0; others NR
Arai 2015 [55]	Median 26 cycles (range 2–84)	NR	None	Disease progression 39; catheter- or procedure-related complication 12; refusal 4; not stated 22
Goi 2015 [62]	3–44 infusions per patient	NR (median survival 9 months)	NR	Toxicity 2; others NR
Kusano 2017 [51]	Planned at least 6 months; median compliance index 0.61	Mean 6 years (whole trial)	Device and compliance data missing for seven of 44	Discontinued seven of 37: haematological toxicity 2, non-haematological toxicity 3, technical problem 1, other 1
Sato 2018 [56]	Median 4 cycles (range 1–11)	NR	None	NR
Kedra 2023 [63]	Median 7 cycles (range 3–22)	Median 7.9 months (range 1.4–15.2)	None; one device removed for infection and re-implanted (timing inconsistent in the source)	NR (all dysfunctions corrected; one right hepatectomy; five deaths)

Counts are patients unless stated. Observation period is follow-up as defined by each source study. “Not visible” indicates a cohort assembled from patients who received HAI, so that patients implanted but never treated cannot be identified. Nine patients in Deschamps 2011 were derived by subtraction (65 implanted, 56 analysed). NR, not reported. Abbreviations: HAI, hepatic arterial infusion.

**Table 5:** Device-related complications of percutaneous HAI ports: frequency, mechanism, prevention and management.

Complication	Frequency in the Included Percutaneous Series	Mechanism	Prevention and Management
Primary implantation failure	2/278 patients (0.7%), both from coeliac trunk stenosis [34]	Unfavourable arterial anatomy	CT angiography to plan the access route; alternative femoral or subclavian access [28,36]
Catheter dislodgement or tip migration	0–19.5% of patients; pooled 5.8% (Table 2)	Catheter not anchored at the point of arterial entry	Side-hole catheter with the tip fixed in the gastroduodenal artery by coils, with or without NBCA [71,72,73]; repositioning or exchange under fluoroscopy, usually without ending HAI [53,61,63]
Hepatic arterial occlusion or thrombosis	0–7.3% of patients; pooled 3.4% (Table 2); 5/10 with a conventional end-hole catheter in one series [58]	Intimal injury and disturbed flow at the catheter tip; drug toxicity	Side-hole placement with tip fixation [58,71]; heparinised flushing after each infusion [55,57]; thrombolysis, although occlusion often ends HAI [34,53]
Extrahepatic perfusion or drug maldistribution	20 events in 56 patients [34]; collateral supply in 3/42 and 7/55 patients [52,59]; gastroduodenal ulcer 2/135 [58]	Unembolised gastroduodenal, right gastric or aberrant branches; collateral development	Embolisation of the gastroduodenal, right gastric and aberrant arteries at placement [36,37]; angiographic or CT check of perfusion through the port [34,53]; re-embolisation, after which HAI usually continued [52,59]
Incomplete hepatic perfusion	Six events in 56 patients [34]	Variant hepatic arteries outside the catheter territory	Coil embolisation of variant arteries to redistribute flow through intrahepatic collaterals [66]; revision [34]
Port or catheter infection	2/56 to 4/54 patients [34,61]; 1/14 [60]; three events in 41 patients [53]	Contamination during repeated port access	Aseptic port access; debridement, or explantation and re-implantation [61,63]
Access-site bleeding	3/56 patients [34]	Arterial puncture	Ultrasound-guided puncture [63]; local compression
Leakage, disconnection or kinking	Leakage or extravasation five and disconnection or rupture five events in 41 patients [53]; disconnection 2/14 [63]	Mechanical failure at the catheter–port junction	Secure connection and port fixation; endovascular or pocket revision [53,63]
Cerebral infarction	1/25 [54]; 3/135 [58]	Embolism during subclavian or axillary access	Femoral access as an alternative route [28]
Hepatic artery dissection	Three events in 41 patients [53]	Guidewire or catheter manipulation	Careful catheter technique; reported more often after surgical placement [34]

Frequencies are from the percutaneous arms of the included studies and are given as patients unless stated as events; ranges and pooled values refer to the primary analyses in Table 2. Abbreviations: CT, computed tomography; HAI, hepatic arterial infusion; NBCA, n-butyl cyanoacrylate.

**Table 6:** Revision, patency and device durability by study.

Study	Technique	Revision/Reintervention	Secondary (Definitive) Device Failure	Patency/Functional Duration
Hildebrandt 2007 [53]	Percutaneous vs. surgical	23/41 (56%) vs. 4/40 (10%) (*p* < 0.001) *	7/41 (17%) vs. 20/40 (50%) (*p* < 0.001) *	Mean 19 vs. 14 mo (*p* = 0.01) *; 77% vs. 50% functional at 12 mo (*p* < 0.01) *
Oberfield 2004 [40]	Percutaneous vs. surgical	60% vs. 14% displacement requiring reinsertion † (*p* < 0.001) *	NR	NR
Seki 2008 [58]	Percutaneous technique variants	NR as an aggregate rate	30/135 (22.2%)	1-yr hepatic arterial patency 76.3–95.0% across the three side-hole variants vs. 38.9% for conventional end-hole (*p* ≤ 0.0014 each) *; 1-yr catheter stability 95.2–96.7% vs. 42.9% *; median time to discontinuation 11.7–14.4 vs. 3.2 mo *
Nishiofuku 2010 [59]	Percutaneous, fixed tip	7/55 (12.7%) re-embolised for collateral supply, HAI continued in all	4/55 (7.3%)	Occlusion at 1.2–8.5 mo; median 12 infusion courses (range 1–58) over a median 3.3 mo
Sameshima 2007 [52]	Percutaneous, fixed tip	3/42 (7.1%) collateral embolisation, treatment restarted in all	3/42 (7.1%)	Occlusions after 18 and 29 infusions; mean 36 treatments per patient (range 10–98)
Deschamps 2011 [34]	Percutaneous vs. surgical	33/56 (59%) vs. 13/55 (24%)	Discontinuation linked to the port–catheter 12/56 (21%) vs. 19/55 (34%) (*p* = 0.12)	Primary functionality 4.80 vs. 4.82 courses (*p* = 0.99); after revision 9.18 vs. 5.95 (*p* = 0.004) *; gain from revision 4.38 vs. 1.13 courses (*p* = 0.0006) *
Kusano 2017 [51]	Radiologically placed (HAI arm of RCT)	NR	1/37 (2.7%) discontinued for a technical problem	Cumulative hepatic arterial catheterisation failure 14.8% at 3 mo and 16.7% at 6 mo
Kedra 2023 [63]	Percutaneous, modified catheter	All seven dysfunctions corrected; one device removed for infection and re-implanted (interval given as 2 and as 3 weeks in the source)	0/14	0.34 dysfunctions and 0.082 complications per 100 catheter-days over a median follow-up of 7.9 mo

† Oberfield 2004 reports catheter displacement requiring reinsertion, not formally labelled revision in the source text; presented here as the closest available proxy. NR, not reported. * Statistically significant (*p* < 0.05) in the source study. Abbreviations: mo, months; yr, year.

**Table 7:** Oncological outcomes by study.

Study	Setting/Line of Therapy	*n*	Response Rate	Median OS	Comment
Oberfield 2004 [40]	Unresectable; 74% of the percutaneous arm and 41% of the surgical arm previously treated systemically (*p* = 0.002)	42 vs. 58	48% vs. 34%	10.6 vs. 13.0 mo	*p* = 0.22 (response); *p* = 0.39 (OS)
Sameshima 2007 [52]	After resection of primary, first-line HAI	42	57.1%	29.1 mo	Single-arm; MST 50.1 mo in pN0 vs. 23.2 mo in pN1–2
Iguchi 2008 [57]	Synchronous unresectable, preoperative	21	Not assessed (liver function was the endpoint)	309 d overall; 386 d in the 19 who underwent resection of the primary	19/21 (90.5%) proceeded to resection of the primary colorectal tumour
Seki 2008 [58]	Unresectable	135	63.6–79.2% (side-hole methods) vs. 50.0% (conventional)	21.1–23.1 vs. 13.1 mo (*p* ≤ 0.0146) *	Comparison is between percutaneous technique variants, not vs. surgical
Nishiofuku 2010 [59]	Refractory to three drugs, salvage	55	18.2% (disease control 70.9%)	6.7 mo	Median PFS 2.8 mo; median hepatic PFS 4.6 mo
Deschamps 2011 [34]	Mixed unresectable and adjuvant	67 vs. 59	Not evaluated	Not evaluated	Response and survival explicitly not assessed in the source study
Lee 2011 [60]	Refractory, salvage	14 (10 eval.)	7% (disease control 50%)	10.7 mo	Median time to progression 4.3 mo
Arai 2012 [54]	Unresectable liver-limited, first-line with systemic irinotecan	25	72% (hepatic)	49.8 mo	Prospective phase I/II; median follow-up 55.0 mo
Shi 2015 [61]	Unresectable synchronous, primary in situ, conversion intent	54	100% hepatic response (CR 18.5%)	3-yr OS 76% if resected (*n* = 38) vs. 15% if not (*n* = 16) (*p* < 0.0001) *	38/54 (70.4%) converted to resectable
Arai 2015 [55]	Primary resected, liver metastases untreated	77	42.9% (disease control 79.2%)	561 d	Median PFS 204 d; prospective multi-institutional phase II
Goi 2015 [62]	Chemotherapy-refractory, salvage	10	30% (disease control 70%)	9 mo	Single-centre case series
Kusano 2017 [51]	Adjuvant after curative resection	44 (HAI)	Not applicable	5-yr OS 34.9% (HAI) vs. 59.0% (systemic), *p* = 0.146	5-yr RFS 20.4% vs. 44.1% (*p* = 0.038) *; trial closed at 91 of a planned 300 patients
Sato 2018 [56]	Refractory, salvage	13 (10 at RD)	10% total lesions; 20% liver lesions	6.9 mo	6-month survival 53.3%
Kedra 2023 [63]	Liver-dominant, mostly heavily pretreated	14	CR 7.1%, PR 21.4%, SD 28.6%	7.7 mo	Median hepatic PFS 6.1 mo; median overall PFS 4.8 mo

Abbreviations: mo, months; OS, overall survival; PFS, progression-free survival; RFS, relapse-free survival. Response rates are as defined and reported by each source study, and are not comparable across differing lines of therapy.

## Data Availability

The data extracted for this systematic review are available within the article and its Supplementary Materials (Supplementary Materials S1 and S2, Tables S1 and S2), including the extracted data and analysis code. Further extraction forms are available from the corresponding author upon reasonable request.

## Supplementary Materials

The following supporting information can be downloaded at: https://www.mdpi.com/article/10.3390/cancers18213495/s1, Supplementary Material S1: Search strategy; Table S1: Risk-of-bias assessments: device-outcome domains at item level for all fifteen studies (Table S1a), Cochrane RoB 2 for the randomised trial (Table S1b) and the Newcastle–Ottawa Scale, for completeness (Table S1c); Table S2a: Outcome-specific, patient-level data extracted for the meta-analyses; Table S2b: Full output of the analysis code: crude pooled proportions with exact Clopper–Pearson 95% CIs, Freeman–Tukey/DerSimonian–Laird and binomial GLMM results; Supplementary Material S2: Analysis code (R 4.3.3; meta 8.0-1, metafor 4.4-0, lme4 1.1-35.1); data file HAI_extraction_S3.csv; code file HAI_meta_analysis_R2.R.
