# Supplementary material for: Percutaneous Insertion of Hepatic Arterial Infusion Ports by Interventional Radiology for Colorectal Liver Metastases
Source: Cancers (Basel). 2026 Oct 29;18(21):3495. doi: 10.3390/cancers18213495 (PMC13762243; doi:10.3390/cancers18213495)
Supplement: Supplementary file 1 [file cancers-18-03495-s001.zip › cancers-4611716-supplementary.pdf]

Supplementary Material

Supplementary Material S1. Search strategy.

- Databases: PubMed, WebOfScience, Scopus, Embase, Cochrane
- Search Window: 1966-2026; records published before 2000 were excluded at screening (publication-date limit)
- Date of Search: 30th June 2026

Scopus: TITLE-ABS (("hepatic artery infusion" OR "hepatic arterial infusion" OR HAI) AND (colorectal OR colon OR rectal OR CRLM OR "colorectal liver metastases") AND (pump* OR catheter* OR port* OR implant* OR device* OR "delivery system"))

PubMed: ("hepatic artery infusion"[Title/Abstract] OR "hepatic arterial infusion"[Title/Abstract] OR HAI[Title/Abstract]) AND (colorectal[Title/Abstract] OR colon[Title/Abstract] OR rectal[Title/Abstract] OR CRLM[Title/Abstract] OR "colorectal liver metastases"[Title/Abstract]) AND (pump*[Title/Abstract] OR catheter*[Title/Abstract] OR port*[Title/Abstract] OR implant*[Title/Abstract] OR device*[Title/Abstract] OR "delivery system"[Title/Abstract])

WebOfScience: TS=(("hepatic artery infusion" OR "hepatic arterial infusion" OR HAI) AND (colorectal OR colon OR rectal OR CRLM OR "colorectal liver metastases") AND (pump* OR catheter* OR port* OR implant* OR device* OR "delivery system"))

Embase: ('hepatic artery infusion':ti,ab OR 'hepatic arterial infusion':ti,ab OR HAI:ti,ab) AND (colorectal:ti,ab OR colon:ti,ab OR rectal:ti,ab OR CRLM:ti,ab OR 'colorectal liver metastases':ti,ab) AND (pump*:ti,ab OR catheter*:ti,ab OR port*:ti,ab OR implant*:ti,ab OR device*:ti,ab OR 'delivery system':ti,ab)

Cochrane: ("hepatic artery infusion":ti,ab OR "hepatic arterial infusion":ti,ab OR HAI:ti,ab) AND (colorectal:ti,ab OR colon:ti,ab OR rectal:ti,ab OR CRLM:ti,ab OR "colorectal liver metastases":ti,ab) AND (pump*:ti,ab OR catheter*:ti,ab OR port*:ti,ab OR implant*:ti,ab OR device*:ti,ab OR "delivery system":ti,ab)

- Citation searching: the reference lists of the included full texts and of the percutaneous technique literature were hand-searched. Two reports identified in this way could not be obtained in full text and are shown separately in the PRISMA flow diagram (Figure 1). Citation and manual searching also identified four reports not retrieved by the database search: one included study (Deschamps 2011, which describes the treatment as intra-arterial hepatic chemotherapy rather than hepatic arterial infusion), one overlapping cohort (Deschamps 2010) and the two reports whose full text could not be obtained. These are shown in the “other methods” arm of the corrected PRISMA flow diagram (Figure 1).

Supplementary Table S1. Risk-of-bias assessments: device-outcome domains at item level for all fifteen studies (Table S1a), Cochrane RoB 2 for the

randomised trial (Table S1b) and the Newcastle–Ottawa Scale, for completeness (Table S1c)

Table S1a. Risk of bias in the device outcomes, judged at item level for the percutaneous arm of each study

**Study**	**Patient selection**	**Completeness of data**	**Outcome ascertainment**	**Selective reporting**	**Overall**
Oberfield 2004 [40]	High: retrospective 1970-1995 series; previously reported patients excluded; 12/42 had crossed over from surgical placement	Moderate: evaluability rules not applied transparently; internal count inconsistencies	Moderate: catheter angiogram every 2 months; no device definitions or grading	Moderate: device complications a secondary comparison	High
Sameshima 2007 [52]	Moderate: explicit criteria; consecutive enrolment and design not stated	Low: all 42 patients in the device outcomes	Moderate: CT arteriography through the port after every 10 infusions; catheter failure undefined	Moderate: safety and efficacy aim; device outcomes not prespecified	Moderate
Hildebrandt 2007 [53]	Moderate: prospective phase II with explicit criteria; first 41 colorectal patients of a trial in liver-confined cancers	Low: all 41 accounted for, including reasons for stopping HAI	Low: DSA before every course, with scintigraphy; no severity grading	Low: device complications and port duration were the primary objective	Moderate
Iguchi 2008 [57]	High: retrospective; 21 of 212 port recipients selected for preoperative HAI	Moderate: all 21 accounted for; after resection of the primary colorectal tumour, the number continuing HAI and their device outcomes not reported	High: port imaging reported only within days of implantation	High: aim was liver function before resection of the primary; later device outcomes not reported	High
Seki 2008 [58]	Moderate: retrospective but sequential enrolment; technique assigned by period	Moderate: 18 of 135 not accounted for in reasons for stopping HAI	Moderate: CT arteriography and DSA 1-10 days after placement and every 2-3 months; patency and stability defined	Low: patency and catheter stability were stated objectives	Moderate
Nishiofuku 2010 [59]	Moderate: four-centre retrospective;	Moderate: cohort defined by receipt	Moderate: CT arteriography	Moderate: efficacy and tolerability	Moderate

Study	Patient selection	Completeness of data	Outcome ascertainment	Selective reporting	Overall
	explicit criteria; consecutiveness not stated	of HAI, so implanted but untreated patients are not visible	through the port every 1-2 months; device failure undefined	aims; device events incidental	
Deschamps 2011 [34]	Low: 126 consecutive patients; complete percutaneous series	Moderate: 9 of 65 successfully implanted percutaneous patients treated elsewhere and not analysed	Low: DSA through the port before every course; complications listed	Low: implantation success, functionality and complications were stated endpoints	Moderate
Lee 2011 [60]	Moderate: all patients receiving HAI in 2004-2009, from a prospectively maintained registry, analysed retrospectively	Moderate: cohort defined by receipt of HAI; inconsistent reporting of one displacement	High: no scheduled device surveillance; device events neither defined nor graded	Moderate: efficacy and safety aims; device events incidental	High
Arai 2012 [54]	Low: prospective registered multicentre phase I/II; explicit criteria	Low: all 25 implanted patients accounted for, including 3 not starting protocol treatment	Low: DSA and CT angiography through the port before each 4-weekly cycle	High: device events during HAI not reported beyond one placement-related stroke; post-protocol treatment not recorded	High
Shi 2015 [61]	High: 54 of 223 consecutive stage IV patients selected by a multidisciplinary team	Moderate: all 54 reported; denominator for total HAI exposure unclear	High: no scheduled device surveillance described; no device definitions	Moderate: resection rate was the main endpoint	High
Arai 2015 [55]	Low: prospective multi-institutional phase II; explicit criteria	Low: all 77 patients evaluable for safety	Moderate: CT angiography every 3 months; catheter complications undefined and ungraded	Moderate: device outcomes within a secondary safety endpoint; 19% complication rate given without a numerator	Moderate
Goi 2015 [62]	High: 10 patients "selected"; design and eligible denominator not stated	High: no device outcome reported	High: angiography at placement only	High: device outcomes not an endpoint	High

Study	Patient selection	Completeness of data	Outcome ascertainment	Selective reporting	Overall
Kusano 2017 [51]	Low: randomised trial with explicit criteria (hepatic arterial infusion arm)	High: device and compliance data for 37 of 44 analysed patients; losses unexplained	High: angiography at 3 and 6 months only; catheter failure undefined	Moderate: device failure not a prespecified endpoint	High
Sato 2018 [56]	Low: prospective two-centre phase I/II; explicit criteria	Low: all 13 patients accounted for	High: DSA and CT angiography before treatment; repeat surveillance schedule not stated	High: device findings mentioned only in the discussion	High
Kedra 2023 [63]	Low: 14 consecutive patients; explicit criteria	Low: all patients and procedures reported (minor denominator inconsistencies)	Low: DSA through the port before the first course and every 4 weeks; complications defined and graded (Clavien-Dindo)	Low: feasibility and safety of the technique were the stated objective	Low

Domains adapted from the JBI critical appraisal checklist for case series and the corresponding ROBINS-I domains (main text, Section 2.4), judged for the device outcomes of the percutaneous arm only. Patient selection: low, consecutive or complete prospective inclusion with explicit criteria; moderate, explicit criteria without stated consecutive inclusion, or a subset of a trial; high, a selected subset of a larger series or no stated criteria. Completeness: low, every implanted patient accounted for; moderate, cohort defined by receipt of HAI or minor unexplained gaps; high, substantial unexplained losses or no device data. Ascertainment: low, port angiography or CT angiography before every course or at least four-weekly; moderate, longer scheduled intervals; high, imaging only at implantation, at fixed distant time points, or when a problem was suspected. Selective reporting: low, device outcomes a stated objective; moderate, device outcomes within secondary safety reporting; high, incidental or absent. Overall, the highest risk in any domain. Abbreviations: CT, computed tomography; DSA, digital subtraction angiography; HAI, hepatic arterial infusion.

Table S1b. Cochrane RoB 2, hepatic arterial infusion arm of Kusano 2017 [51]

Domain	Judgement	Basis
Randomisation process	Low	Randomisation performed centrally by the JFMC data centre and stratified by primary site, Dukes stage, synchronous or metachronous presentation and number of liver metastases
Deviations from intended interventions	Some concerns	Open-label by necessity, since the two arms differ in route of administration; the trial closed after enrolling 91 of a planned 300 patients
Missing outcome data	Some concerns	Compliance and adverse-event data available for 37 of 44 patients in the hepatic arterial infusion arm, with denominators reported inconsistently between text and tables
Measurement of the	Some	Survival and relapse-free survival ascertained objectively; catheter-

Domain	Judgement	Basis
outcome	concerns	related complications reported as an approximate proportion without a pre-specified definition or outcome assessor blinding
Selection of the reported result	Some concerns	Device outcomes reported without pre-specified definitions, in a trial stopped well short of its planned sample size
Overall (device outcomes)	Some concerns	Reliable for the randomised survival comparison; the device outcomes used in this review are secondary, incompletely defined and based on a reduced denominator

The randomised comparison of hepatic arterial versus systemic chemotherapy is not an outcome of this review; the assessment above relates to the device outcomes extracted from the hepatic arterial infusion arm.

Table S1c. Newcastle-Ottawa Scale, fourteen non-randomised studies (reported for completeness; not used to interpret the evidence)

Study	Selection (/4)	Comparability (/2)	Outcome (/3)	Total (/9)	Judgement
Oberfield 2004 [40]	4	1	3	8	Good
Hildebrandt 2007 [53]	4	1	3	8	Good
Deschamps 2011 [34]	4	1	3	8	Good
Arai 2012 [54]	4	0	3	7	Good
Arai 2015 [55]	4	0	3	7	Good
Sameshima 2007 [52]	3	0	3	6	Fair
Iguchi 2008 [57]	3	0	3	6	Fair
Seki 2008 [58]	3	0	3	6	Fair
Nishiofuku 2010 [59]	3	0	3	6	Fair
Lee 2011 [60]	3	0	3	6	Fair
Shi 2015 [61]	3	0	3	6	Fair
Sato 2018 [56]	3	0	3	6	Fair
Kedra 2023 [63]	3	0	2	5	Fair
Goi 2015 [62]	2	0	2	4	Poor

Comparability was scored 0 where no technique-stratified comparator group was analysed within the study (all studies except Oberfield 2004, Hildebrandt 2007 and Deschamps 2011). Good ≥7, Fair 5-6, Poor ≤4. Abbreviations: NOS, Newcastle-Ottawa Scale. The Newcastle-Ottawa Scale rewards a comparator group and adjustment for confounding, which do not bear on the reliability of single-arm device outcomes; it is therefore superseded by Table S2a for interpretation.

Supplementary Table S2a. Outcome-specific, patient-level data extracted for the meta-analyses.

Study	Outcome	Events	Patients	Analysis	Source and denominator note
Sameshima 2007 [52]	Technical success	42	42	Primary	stated
Hildebrandt 2007 [53]	Technical success	41	41	Primary	stated
Iguchi 2008 [57]	Technical success	21	21	Primary	stated
Nishiofuku 2010 [59]	Technical success	55	55	Primary	stated

Study	Outcome	Events	Patients	Analysis	Source and denominator note
Deschamps 2011 [34]	Technical success	65	67	Primary	stated; strictest definition
Arai 2012 [54]	Technical success	25	25	Primary	stated
Sato 2018 [56]	Technical success	13	13	Primary	stated
Kedra 2023 [63]	Technical success	14	14	Primary	stated per procedure (15/15); first implantation succeeded in all 14 patients
Seki 2008 [58]	Technical success	135	135	Sensitivity	stated; surgically exposed access vessel
Sameshima 2007 [52]	Definitive failure	3	42	Primary	patients
Hildebrandt 2007 [53]	Definitive failure	7	41	Primary	patients with treatment-limiting (terminal) device complication
Iguchi 2008 [57]	Definitive failure	0	21	Primary	patients
Nishiofuku 2010 [59]	Definitive failure	4	55	Primary	patients
Deschamps 2011 [34]	Definitive failure	12	56	Primary	patients who began HAI; discontinuation linked to port-catheter complication
Lee 2011 [60]	Definitive failure	1	14	Primary	patients
Shi 2015 [61]	Definitive failure	0	54	Primary	patients
Arai 2015 [55]	Definitive failure	12	77	Primary	patients
Kedra 2023 [63]	Definitive failure	0	14	Primary	patients
Sato 2018 [56]	Definitive failure	0	13	Sensitivity only	no reasons for stopping HAI reported; zero count rests on the statement that no tip migration or perfusion abnormality was observed, so sensitivity analysis only
Seki 2008 [58]	Definitive failure	30	135	Sensitivity	patients; all four technique arms
Seki 2008 (side-hole arms) [58]	Definitive failure	22	125	Sensitivity	patients; three side-hole arms
Arai 2012 [54]	Definitive failure	1	25	Sensitivity	HAI never initiated after cerebral infarction attributed to catheter placement; device events during HAI not reported
Lee 2011 [60] (alternative count)	Definitive failure	2	14	Sensitivity	Results list catheter thrombosis as the only device-related reason for stopping; toxicity section also reports a displacement leading to catheter removal and discontinuation of HAI
Sameshima 2007 [52]	Dislodgement / migration	1	42	Primary	patients
Iguchi 2008 [57]	Dislodgement / migration	0	21	Primary	patients
Lee 2011 [60]	Dislodgement / migration	1	14	Primary	patients
Shi 2015 [61]	Dislodgement / migration	2	54	Primary	patients
Sato 2018 [56]	Dislodgement / migration	0	13	Primary	patients
Kedra 2023 [63]	Dislodgement / migration	1	14	Primary	single event
Seki 2008 [58]	Dislodgement / migration	11	135	Sensitivity	patients

Study	Outcome	Events	Patients	Analysis	Source and denominator note
Deschamps 2011 [34]	Dislodgement / migration	8	56	Primary	Table 2 lists 8 migration events; Results give the reasons for revision of the 33 revised patients (extrahepatic perfusion 19, incomplete perfusion 5, occlusion 1, migration 8; sum 33), so the 8 events occurred in exactly 8 distinct patients
Hildebrandt 2007 [53]	Dislodgement / migration	8	41	Primary	Table 3 lists 8 dislocation events; Results give the reason for first revision of the 23 revised patients (dislocation 8, leakage 5, thrombus or embolism 5, disconnection 3, occlusion 1, infection 1; sum 23), so the 8 events occurred in exactly 8 distinct patients
Sameshima 2007 [52]	Arterial occlusion / thrombosis	2	42	Primary	patients
Iguchi 2008 [57]	Arterial occlusion / thrombosis	0	21	Primary	patients
Nishiofuku 2010 [59]	Arterial occlusion / thrombosis	4	55	Primary	patients
Deschamps 2011 [34]	Arterial occlusion / thrombosis	1	56	Primary	single event
Seki 2008 [58]	Arterial occlusion / thrombosis	25	135	Sensitivity	patients; all arms
Seki 2008 (side-hole arms) [58]	Arterial occlusion / thrombosis	20	125	Sensitivity	patients; side-hole arms
Hildebrandt 2007 [53]	Arterial occlusion / thrombosis	7	41	Sensitivity	Events (7 total thromboses among 39 events in 26 patients); unique patients not derivable (5-7); sensitivity only

Counts are patients unless the note states otherwise. Primary analyses include only fully percutaneous implanted ports; Seki 2008 (surgically exposed access artery) and event counts for which the number of patients affected could not be determined enter sensitivity analyses only. Technical success was not inferred for any study that did not state it.

Supplementary Table S2b. Full output of the analysis code: crude pooled proportions with exact Clopper–Pearson 95% CIs, Freeman–Tukey/DerSimonian–Laird and binomial GLMM results.

Analysis	k	Events/N	Crude % (exact 95% CI)	FT pooled % (95% CI)	I² (%)	GLMM % (95% CI)	GLMM tau²
Technical success: primary (fully percutaneous implanted ports)	8	276/278	99.3 (97.4-99.9)	99.9 (98.7-100.0)	0.0	99.6 (88.6-100.0)	0.916
TS sensitivity: adding Seki 2008 (surgically exposed access vessel)	9	411/413	99.5 (98.3-99.9)	100.0 (99.4-100.0)	0.0	99.9 (85.0-100.0)	2.64
TS subgroup: prospective	4	121/121	100.0 (97.0-100.0)	100.0 (98.5-100.0)	0.0	NE	NE

Analysis	k	Events/N	Crude % (exact 95% CI)	FT pooled % (95% CI)	I² (%)	GLMM % (95% CI)	GLMM tau²
TS subgroup: retrospective	4	155/157	98.7 (95.5-99.8)	99.6 (97.2-100.0)	0.0	98.7 (95.1-99.7)	0
TS subgroup: enrolment began before 2005	5	194/196	99.0 (96.4-99.9)	99.6 (97.6-100.0)	0.0	99.1 (90.2-99.9)	0.187
TS subgroup: enrolment began 2005 or later	3	82/82	100.0 (95.6-100.0)	100.0 (98.3-100.0)	0.0	NE	NE
Definitive failure: primary	9	39/374	10.4 (7.5-14.0)	6.9 (2.1-13.7)	74.5	6.9 (3.0-15.2)	0.858
DF sensitivity: adding Seki 2008, all arms	10	69/509	13.6 (10.7-16.8)	8.4 (3.2-15.4)	79.7	8.2 (3.9-16.6)	0.862
DF sensitivity: adding Seki 2008, side-hole arms only	10	61/499	12.2 (9.5-15.4)	8.1 (3.3-14.4)	75.8	8.2 (4.1-16.0)	0.71
DF sensitivity: excluding Iguchi 2008	8	39/353	11.0 (8.0-14.8)	8.1 (2.8-15.5)	74.6	8.3 (4.0-16.4)	0.62
DF sensitivity: adding Sato 2018 (0/13; no device abnormality reported, reasons for stopping not reported)	10	39/387	10.1 (7.3-13.5)	6.2 (1.7-12.4)	72.7	6.1 (2.5-14.3)	1
DF sensitivity: adding Arai 2012 (1/25 never began HAI after placement-related cerebral infarction)	10	40/399	10.0 (7.3-13.4)	6.7 (2.2-12.8)	71.7	6.6 (3.0-13.9)	0.806
DF sensitivity: Lee 2011 counted as 2/14 (displacement also reported as ending HAI)	9	40/374	10.7 (7.8-14.3)	7.4 (2.4-14.4)	75.0	7.5 (3.4-15.7)	0.792
DF sensitivity: restricted to studies at low or moderate overall risk of bias (Table S1a)	6	38/285	13.3 (9.6-17.8)	11.6 (6.3-18.0)	51.6	12.6 (8.1-19.0)	0.1
Dislodgement: primary (patient-level counts)	8	21/255	8.2 (5.2-12.3)	5.8 (1.6-11.6)	53.6	6.0 (2.5-13.4)	0.606
DIS sensitivity: adding Seki 2008	9	32/390	8.2 (5.7-11.4)	6.2 (2.7-10.8)	47.1	6.8 (3.6-12.7)	0.358
DIS sensitivity: excluding the two counts derived from revision data (Deschamps 2011, Hildebrandt 2007)	6	5/158	3.2 (1.0-7.2)	2.1 (0.1-5.7)	0.0	3.2 (1.3-7.4)	0
DIS subgroup: port angiography before every course or every 4 weeks	3	17/111	15.3 (9.2-23.4)	14.8 (8.4-22.4)	0.0	15.3 (9.7-23.3)	0
DIS subgroup: less frequent or unspecified device surveillance	5	4/144	2.8 (0.8-7.0)	1.8 (0.0-5.5)	0.0	2.8 (1.0-7.2)	0

Analysis	k	Events/N	Crude % (exact 95% CI)	FT pooled % (95% CI)	I^2 (%)	GLMM % (95% CI)	GLMM tau^2
Occlusion: primary (patient-level counts)	4	7/174	4.0 (1.6-8.1)	3.4 (0.9-7.0)	0.0	4.0 (1.9-8.2)	0
OCC sensitivity: adding Seki 2008, all arms	5	32/309	10.4 (7.2-14.3)	5.8 (0.8-13.9)	79.7	5.4 (1.9-14.5)	0.797
OCC sensitivity: adding Seki 2008, side-hole arms only	5	27/299	9.0 (6.0-12.9)	5.5 (1.0-12.4)	73.0	5.4 (2.0-13.6)	0.611
OCC sensitivity: adding Hildebrandt 2007 event count (7 events; unique patients 5-7)	5	14/215	6.5 (3.6-10.7)	5.2 (1.1-11.3)	58.3	5.3 (2.1-12.8)	0.509

FT, Freeman-Tukey double arcsine; GLMM, binomial-normal random-intercept logistic model fitted by maximum likelihood; NE, not estimable (every study at 100%, where the maximum-likelihood GLMM cannot be fitted). All other GLMMs converged without warnings (convergence log in the output of Supplementary Material S2). Between-subgroup tests: technical success by design Q=0.52, df=1, p=0.469; by enrolment era Q=0.26, df=1, p=0.612; dislodgement by device surveillance Q=12.22, df=1, p<0.001. Overall risk of bias as in Table S2a.

Supplementary Material S2. Analysis code (R 4.3.3; meta 8.0-1, metafor 4.4-0, lme4 1.1-35.1). The script reads the data in Table S2a (file HAI_extraction_S3.csv, supplied with the code) and reproduces every estimate in Table 2 and Table S2b.

```
## Supplementary Material S2 - analysis code
## Percutaneous HAI port insertion for CRLM: single-arm proportion meta-analyses
## Primary model (prespecified): random effects, Freeman-Tukey double arcsine (PFT), DerSimonian-Laird
tau^2,
##   back-transformed with the harmonic mean of study sizes.
## Alternative model (first revision): binomial-normal random-intercept logistic GLMM (maximum likelihood),
##   which models within-study binomial variation exactly and needs no transformation at 0 or 100%.
## Second revision: (i) crude pooled proportions with exact Clopper-Pearson 95% CIs reported for every
analysis;
##   (ii) every warning raised while fitting the GLMM is captured and reported (convergence log);
##    (iii) Sato 2018 moved from the primary definitive-failure analysis to a sensitivity analysis;
sensitivity
##   analyses added for definitive failure (Sato 2018; Arai 2012; Lee 2011 alternative count);
##   (iv) dislodgement re-analysed by device-surveillance protocol; (v) definitive failure restricted to
studies
##   at low or moderate overall risk of bias on the device-outcome assessment (Supplementary Table S1a).
## Counts are patient-level numerators/denominators as reported in each source study (Supplementary Table
S2a).
## Input file: HAI_extraction_S3.csv (supplied with this script).

suppressPackageStartupMessages(library(meta))
cat("R:", R.version.string, "| meta:", as.character(packageVersion("meta")),
    "| metafor:", as.character(packageVersion("metafor")),
    "| lme4:", as.character(packageVersion("lme4")), "\n\n")

d <- read.csv("HAI_extraction_S3.csv", stringsAsFactors = FALSE)
glmm_log <- list()

run <- function(label, outcome, studies) {
  x <- d[d$outcome == outcome & d$study %in% studies, ]
  stopifnot(nrow(x) == length(studies))
```

```
  ft <- metaprop(event, n, studlab = study, data = x, sm = "PFT",
               method = "Inverse", method.tau = "DL", common = FALSE, random = TRUE)
  warns <- character(0)
  gl <- withCallingHandlers(
    tryCatch(metaprop(event, n, studlab = study, data = x, sm = "PLOGIT",
                    method = "GLMM", common = FALSE, random = TRUE),
           error = function(e) { warns <<- c(warns, paste("ERROR:", conditionMessage(e))); NULL }),
    warning = function(w) { warns <<- c(warns, conditionMessage(w)); invokeRestart("muffleWarning") })
  glmm_log[[label]] <<- if (length(warns)) unique(warns) else "no warnings; converged"
  bt <- function(m) {
    if (is.null(m)) return(rep(NA, 3))
    v <- c(m$TE.random, m$lower.random, m$upper.random)
    if (m$sm == "PFT") 100 * meta:::asin2p(v, rep(1 / mean(1 / m$n), 3)) else 100 * plogis(v)
  }
  f <- bt(ft); g <- bt(gl)
  ## GLMM interval not estimable when every study sits at 0% or 100% (no between-study information)
  ## (metafor::rma.glmm cannot fit the ML model in this case; meta returns a degenerate 0-100% interval)
  if (all(x$event == x$n) || all(x$event == 0)) {
    g <- rep(NA, 3)
    glmm_log[[label]] <<- "not estimable: every study at the 0%/100% boundary, ML GLMM cannot be fitted"
  }
  ex <- binom.test(sum(x$event), sum(x$n))$conf.int * 100
  data.frame(analysis = label, k = nrow(x),
           events_N = paste0(sum(x$event), "/", sum(x$n)),
           crude = round(100 * sum(x$event) / sum(x$n), 1),
           crude_lo = round(ex[1], 1), crude_hi = round(ex[2], 1),
           FT_pooled = round(f[1], 1), FT_lo = round(f[2], 1), FT_hi = round(f[3], 1),
           I2 = round(100 * ft$I2, 1), Q = round(ft$Q, 2), Q_p = signif(ft$pval.Q, 2),
           GLMM_pooled = round(g[1], 1), GLMM_lo = round(g[2], 1), GLMM_hi = round(g[3], 1),
           GLMM_tau2 = if (is.null(gl)) NA else signif(gl$tau2, 3),
           GLMM_warnings = length(setdiff(glmm_log[[label]], "no warnings; converged")))
}


core <- c("Sameshima 2007","Hildebrandt 2007","Iguchi 2008","Nishiofuku 2010","Deschamps 2011",
        "Arai 2012","Sato 2018","Kedra 2023")
df_core <- c("Sameshima 2007","Hildebrandt 2007","Iguchi 2008","Nishiofuku 2010","Deschamps 2011",
           "Lee 2011","Shi 2015","Arai 2015","Kedra 2023")
## Sato 2018 reports no reasons for stopping HAI; its 0/13 rests on a statement that no tip migration or
perfusion
## abnormality was observed, so it enters a sensitivity analysis only (second revision)
dis_core  <-  c("Sameshima  2007","Hildebrandt  2007","Iguchi  2008","Deschamps  2011","Lee  2011","Shi
2015","Sato 2018","Kedra 2023")
occ_core <- c("Sameshima 2007","Iguchi 2008","Nishiofuku 2010","Deschamps 2011")
## Device checked through the port by angiography or CT angiography before every course or every 4 weeks
dis_surv <- c("Hildebrandt 2007","Deschamps 2011","Kedra 2023")
## Overall device-outcome risk of bias low or moderate (Supplementary Table S1a)
df_lowmod <- c("Sameshima 2007","Hildebrandt 2007","Nishiofuku 2010","Deschamps 2011","Arai 2015","Kedra
2023")


res <- rbind(
  run("Technical success: primary (fully percutaneous implanted ports)", "TS", core),
  run("TS sensitivity: adding Seki 2008 (surgically exposed access vessel)", "TS", c(core, "Seki 2008")),
  run("TS subgroup: prospective", "TS", c("Sameshima 2007","Hildebrandt 2007","Arai 2012","Sato 2018")),
  run("TS subgroup: retrospective", "TS", c("Iguchi 2008","Nishiofuku 2010","Deschamps 2011","Kedra 2023")),
  run("TS  subgroup:  enrolment  began  before  2005",  "TS",  c("Sameshima  2007","Hildebrandt  2007","Iguchi
2008","Deschamps 2011","Arai 2012")),
  run("TS subgroup: enrolment began 2005 or later", "TS", c("Nishiofuku 2010","Sato 2018","Kedra 2023")),
  run("Definitive failure: primary", "DF", df_core),
  run("DF sensitivity: adding Seki 2008, all arms", "DF", c(df_core, "Seki 2008")),
  run("DF sensitivity: adding Seki 2008, side-hole arms only", "DF", c(df_core, "Seki 2008 side-hole")),
  run("DF sensitivity: excluding Iguchi 2008", "DF", setdiff(df_core, "Iguchi 2008")),
  run("DF  sensitivity:  adding  Sato  2018  (0/13;  no  device  abnormality  reported,  reasons  for  stopping  not
reported)", "DF", c(df_core, "Sato 2018")),
  run("DF sensitivity: adding Arai 2012 (1/25 never began HAI after placement-related cerebral infarction)",
"DF", c(df_core, "Arai 2012")),
  run("DF sensitivity: Lee 2011 counted as 2/14 (displacement also reported as ending HAI)", "DF",
      c(setdiff(df_core, "Lee 2011"), "Lee 2011 alt")),
  run("DF  sensitivity:  restricted  to  studies  at  low  or  moderate  overall  risk  of  bias  (Table  S1a)",  "DF",
df_lowmod),
  run("Dislodgement: primary (patient-level counts)", "DIS", dis_core),
  run("DIS sensitivity: adding Seki 2008", "DIS", c(dis_core, "Seki 2008")),
  run("DIS  sensitivity:  excluding  the  two  counts  derived  from  revision  data  (Deschamps  2011,  Hildebrandt
2007)", "DIS",
      setdiff(dis_core, c("Deschamps 2011", "Hildebrandt 2007"))),
  run("DIS subgroup: port angiography before every course or every 4 weeks", "DIS", dis_surv),
  run("DIS subgroup: less frequent or unspecified device surveillance", "DIS", setdiff(dis_core, dis_surv)),
  run("Occlusion: primary (patient-level counts)", "OCC", occ_core),
  run("OCC sensitivity: adding Seki 2008, all arms", "OCC", c(occ_core, "Seki 2008")),
  run("OCC sensitivity: adding Seki 2008, side-hole arms only", "OCC", c(occ_core, "Seki 2008 side-hole")),
  run("OCC  sensitivity:  adding  Hildebrandt  2007  event  count  (7  events;  unique  patients  5-7)",  "OCC",
c(occ_core, "Hildebrandt 2007"))
)
options(width = 250)
print(res, row.names = FALSE)
write.csv(res, "HAI_meta_results_R2.csv", row.names = FALSE)


## Between-subgroup tests (FT/DL)
x <- d[d$outcome == "TS" & d$study %in% core, ]
```

```
x$design <- ifelse(x$study %in% c("Sameshima 2007","Hildebrandt 2007","Arai 2012","Sato 2018"), "prospective", "retrospective")
x$era <- ifelse(x$study %in% c("Nishiofuku 2010","Sato 2018","Kedra 2023"), "2005+", "pre-2005")
for (s in c("design", "era")) {
  m <- metaprop(event, n, studlab = study, data = x, sm = "PFT", method = "Inverse",
                method.tau = "DL", common = FALSE, subgroup = x[[s]])
  cat(sprintf("\nTS subgroup test by %s: Q = %.2f, df = %d, p = %.3f", s, m$Q.b.random, m$df.Q.b, m$pval.Q.b.random))
}
y <- d[d$outcome == "DIS" & d$study %in% dis_core, ]
y$surv <- ifelse(y$study %in% dis_surv, "per-course/4-weekly", "less frequent")
m <- metaprop(event, n, studlab = study, data = y, sm = "PFT", method = "Inverse",
              method.tau = "DL", common = FALSE, subgroup = y$surv)
cat(sprintf("\nDIS subgroup test by surveillance: Q = %.2f, df = %d, p = %.2g\n", m$Q.b.random, m$df.Q.b, m$pval.Q.b.random))


cat("\nGLMM convergence log (all warnings raised by metafor::rma.glmm / lme4 during fitting):\n")
for (nm in names(glmm_log)) cat(" -", nm, ":", paste(glmm_log[[nm]], collapse = " | "), "\n")
```
